# Physical Activity and Weight Loss Among Adults With Type 2 Diabetes and Overweight or Obesity: A Post Hoc Analysis of the Look AHEAD Trial

**DOI:** 10.1001/jamanetworkopen.2024.0219

**Published:** 2024-02-22

**Authors:** Zihao Huang, Xiaodong Zhuang, Rihua Huang, Menghui Liu, Xinghao Xu, Ziyan Fan, Rongling Dai, Hansheng Li, Zhenyu Xiong, Yue Guo, Qi Liang, Xinxue Liao

**Affiliations:** 1 Department of Rehabilitation Medicine, the First Affiliated Hospital, Sun Yat-Sen University, Guangzhou, China; 2 Department of Cardiology, the First Affiliated Hospital, Sun Yat-Sen University, Guangzhou, China; 3 National Health Commission Key Laboratory of Assisted Circulation, Sun Yat-Sen University, Guangzhou, China; 4 School of Journalism and Communication, Sun Yat-Sen University, Guangzhou, China; 5 School of Critical Studies, University of Glasgow, Glasgow, United Kingdom

## Abstract

**Importance:**

Prior findings from the Look AHEAD trial showed no significant reduction in the risk of cardiovascular events by lifestyle-induced weight loss among individuals with type 2 diabetes (T2D) and overweight or obesity. However, physical activity (PA) may modify the changes in cardiovascular risk associated with weight loss.

**Objective:**

To examine the joint association of weight loss and PA with the risk of adverse cardiovascular events in patients with T2D and overweight or obesity.

**Design, Setting, and Participants:**

This cohort study was a post hoc analysis of the Look AHEAD randomized clinical trial, which compared the cardiovascular effects of weight loss by intensive lifestyle intervention vs diabetes support and education among individuals with T2D and overweight or obesity. The study was conducted from June 2001 to September 2012, and participants were patients in the substudy of accelerometry-measured PA from 8 locations in the United States. Data were analyzed from June to August 2023.

**Exposures:**

Body weight change and accelerometer-derived PA volume across the first 4 years.

**Main Outcomes and Measures:**

The primary outcome was a composite cardiovascular outcome including cardiovascular death, nonfatal myocardial infarction, nonfatal stroke, or hospitalization for angina.

**Results:**

Among a total of 1229 participants (mean [SD] age, 60 [7] years; 533 male [43%]), 333 (27%) achieved and maintained weight loss for the first 4 years. Among the individuals who maintained weight loss, 105 (32%) maintained high PA volume. During a median of 9.5 years of follow-up, 198 participants (16.1%) experienced the primary outcome. Compared with those with low PA volume and no weight loss (105 [15.8%]), maintaining high PA volume and weight loss was associated with a 61% lower risk of the primary end point (hazard ratio, 0.39; 95% CI, 0.19-0.81; *P* = .01). However, there was no significant difference in the risk of the primary end point among those with either weight loss only or high PA only. The multiplicative interaction between weight loss and PA for the risk of cardiovascular events was also significant (*P* for interaction = .01).

**Conclusions and Relevance:**

In this cohort study, maintaining weight loss and higher PA volume was associated with a lower risk of the composite cardiovascular outcome. The findings suggest that the cardiovascular benefits of PA may vary and be enhanced by weight loss among individuals with T2D and overweight or obesity.

## Key Points

**Question:**

Does physical activity (PA) modify the cardiovascular benefits of weight loss in individuals with type 2 diabetes and overweight or obesity?

**Findings:**

In this cohort study of 1229 individuals who participated in the Look AHEAD trial, incorporating high PA volume and weight loss was associated with a 61% of lower risk of adverse cardiovascular events.

**Meaning:**

The findings of this study suggest that the benefits of PA may vary by and be enhanced during weight loss.

## Teaser

This post hoc analysis of the Look AHEAD trial examines the joint association of weight loss and physical activity with the risk of cardiovascular events in patients with type 2 diabetes and overweight or obesity in the United States.

## Introduction

Nearly 10% of cardiovascular events are attributed to type 2 diabetes (T2D).^1^ In 2019, it was estimated that more than 40 million people developed T2D, and the number was projected to exceed 600 million by 2045.^2,3^ Obesity is connected with T2D and shares key pathophysiological mechanisms.^4^ Maintaining a meaningful magnitude of weight loss is considered and recommended as a primary treatment goal for T2D.^3,4,5^ Increasing moderate-to-vigorous physical activity (PA) volume is a beneficial lifestyle change and a conventional manner of weight loss,^3,5,6,7^ and higher PA volume or weight loss has beem associated with a lower risk of cardiovascular events.^8,9,10,11^ However, the evidence supporting lifestyle-induced weight loss and increased PA are classified as level B in guidelines, indicating that the recommendations are based on limited randomized clinical trials (RCTs).^3,5,7,12^ Prior large RCTs, including Look AHEAD (Action for Health in Diabetes), DIRECT, and the Diabetes Prevention Program, attempted to investigate the cardiovascular benefits of weight loss and increased PA volume among individuals with prediabetes or T2D, but no relationship between lifestyle-induced weight loss and cardiovascular benefits has been reported.^13,14,15^ Consequently, additional research is crucial to deepen our knowledge of the outcomes of lifestyle-induced weight loss. Recent studies have indicated that increased PA volume could enhance the metabolic benefits of weight loss,^16,17,18^ but it is unclear whether the association between weight loss and the risk of cardiovascular events differs by PA volumes. Therefore, this study evaluates the interactions of weight loss and PA volumes in association with the risk of cardiovascular events by conducting a post hoc secondary analysis of the Look AHEAD trial among individuals with T2D and overweight or obesity, defined as a body mass index (calculated as weight in kilograms divided by height in meters squared) of 25 or greater (or ≥27 if currently taking insulin).

## Methods

### Study Design and Participants

This cohort study was a post hoc secondary analysis of the Look AHEAD trial (NCT00017953). The detailed design, randomization and masking, and protocol of the Look AHEAD trial have been published previously.^19^ Briefly, the Look AHEAD trial was a multicenter RCT that investigated the cardiovascular benefits of an intensive lifestyle intervention (ILI), aiming for at least 7% weight loss from the baseline body weight, compared with diabetes support and education (DSE).^14,19^ The eligibility, exclusion criteria, and study interventions appear in the eMethods in Supplement 1. The study was approved by the local institutional review boards, and written informed consent was provided by all participants. This study obtained approval from the institutional review boards of all clinical sites and followed the Strengthening the Reporting of Observational Studies in Epidemiology (STROBE) reporting guideline.

A substudy of accelerometry-measured PA was conducted at 8 of the clinical sites, including Baltimore, Maryland; Baton Rouge, Louisiana; Denver, Colorado; Houston, Texas; Memphis, Tennessee; Minneapolis, Minnesota; Philadelphia, Pennsylvania; and Providence, Rhode Island. The 2143 participants engaged in the substudy at baseline were included in this analysis (eFigure 1 in Supplement 1). PA was also measured at year 1 and 4, and for trajectory analysis, 1434 participants with complete accelerometry-measured PA data were included. Participants wearing accelerometry for less than 4 valid days (172 individuals) and those with missing covariates (33 participants) were also excluded.^8^ Overall, 1229 participants were included in the analysis and have similar baseline characteristics as those excluded (eTable 1 in Supplement 1).

### Weight Loss

Weight loss was the primary intervention goal in the Look AHEAD trial, but a noticeable weight regain was observed after the first year of the intervention. Consequently, a 4-year weight loss percentage was calculated by subtracting the body weight at year 4 from the baseline weight, then dividing this difference by the baseline weight. Year 4 was chosen because participants received more frequent PA measurements, individual supervision, and group sessions in the first 4 years.

### Accelerometry-Measured PA Volume

Physical activity was assessed by a triaxial accelerometer (RT3 [Stayhealthy]) at baseline, year 1, and year 4. Details and parameters of accelerometry-measured PA have been shown in prior studies.^20,21^ Briefly, a valid wear-day was defined as the accelerometer being worn for at least 10.5 hours within 1 day.^9^ Nonwear time was defined as consecutive 0 count of the accelerometer for more than 60 minutes. Participants were instructed to wear the accelerometer for 7 days, including a weekend day, placing the device vertically at waist at the horizontal level of the anterior iliac spine. PA that lasted for at least 10 consecutive minutes with an intensity greater than 3 metabolic equivalent of tasks (METs) was considered valid moderate-to-vigorous PA.^8,9^ The total PA volume (MET-min/wk) was calculated by summing up the minutes of moderate-to-vigorous PA and multiplying the corresponding intensity (METs).

### Primary and Secondary Outcomes

The primary outcome was the first postrandomization occurrence of a composite cardiovascular outcome including death from cardiovascular causes, nonfatal myocardial infarction, nonfatal stroke, or hospitalization for angina. Three secondary composite cardiovascular outcomes included (1) death from cardiovascular causes, nonfatal myocardial infarction, or nonfatal stroke; (2) death from any cause, nonfatal myocardial infarction, nonfatal stroke, or hospitalization for angina; and (3) death from any cause, nonfatal myocardial infarction, nonfatal stroke, or hospitalization for angina combined with coronary artery bypass grafting, percutaneous coronary intervention, hospitalization for heart failure, carotid endarterectomy, or peripheral vascular disease. The outcomes were adjudicated by a masked outcome committee.

### Statistical Analysis

Descriptive statistics were used to describe baseline characteristics. Continuous variables are reported as mean and SD, and categorical variables are reported as number and percentage. Details of model establishment and parameters were displayed in the eMethods in Supplement 1.

The analyses were conducted within 2 weight loss categories and PA trajectories, respectively, first (eMethods in Supplement 1), and stratified analyses were performed according to the weight loss categories and PA trajectories. Group-based trajectory modeling was used to identify clusters of individuals with similar patterns of longitudinal PA volume in the first 4 years.^22^ The optimal number of trajectory groups and models was determined by the Akaike and bayesian information criterion (BIC), higher mean posterior probability (>0.7), and number of observations in each group (eTable 2 and eFigure 2A in Supplement 1). Finally, 2 distinct trajectories turned out to be the best-fitting model with the most reasonable sample sizes across groups (eFigure 2B in Supplement 1), characterizing low and high PA volume (eTable 3 in Supplement 1). To evaluate the effects of PA, the PA trajectory was included as an independent variable of group stratification.

Cumulative incidences were estimated for each outcome for all categories using the Kaplan-Meier method. Cox proportional hazards regression models were constructed to evaluate the association between weight loss and the risk of cardiovascular outcomes stratified by PA trajectories. Two multivariable models were built to adjust the potential confounders, and the adjusted covariate measurements are displayed in the eMethods in Supplement 1. Model 1 was adjusted for age, sex, and race at baseline. Race was self-reported, with categories consistent with those used in the National Health and Nutrition Examination Study (ie, African American or Black, non-Hispanic; Asian; Hispanic; Pacific Islander; White; and other). Asian, Pacific Islander, and other were collapsed into the other or multiracial group due to small sample sizes. Model 2 was additionally adjusted for history of cardiovascular events, fasting plasma glucose, history of hypertension, insulin use, aspirin use, sedentary time, drinking status, smoking status, triglyceride level, and treatment group at baseline. The variance inflation factors were calculated to check collinearity (eTable 4 in Supplement 1). Multiplicative interaction terms were included in the most-adjusted Cox models to evaluate whether PA modifies the associations between weight loss and each outcome. Restricted cubic splines were constructed to evaluate the association of accumulative mean PA with the risk of each outcome, stratified by weight loss categories.

A joint model was conducted to investigate the isolated and joint association between PA, weight loss, and cardiovascular outcomes over time. Initially, the basic joint model was established comprising (1) a longitudinal analysis of the logarithmic transformation of PA volume + 1, weight loss categories, and time using linear mixed effects with random intercepts and slopes and (2) a Cox model adjusted the other aforementioned covariates. Subsequently, the associations between baseline values, slopes interacting with weight loss categories, and the outcomes were further estimated.^23^ Finally, the linear and nonlinear associations between PA with time were estimated.^23^

Sensitivity analyses were performed according to the American Diabetes Association (ADA) guidelines, which recommend weight loss of at least 5%, and treating weight loss as continuous variables.^5^ Recent studies also indicated any moderate-to-vigorous PA might be beneficial.^24,25,26^ A sensitivity analysis was performed by using the data of any moderate-to-vigorous PA duration of at least 1 minute. Sensitivity analyses were also conducted by using the clustering model with the lowest BIC (model 6 in eTable 2 in Supplement 1), excluding participants who experienced outcomes in the first 4 years and including those participating in any follow-up PA measures. Subgroup analyses for the primary outcome were further conducted by stratifying the treatment group, age, sex and race. A 2-sided *P* < .05 was considered statistically significant. The PA trajectories and joint models were established in R version 4.2.2 (R Project for Statistical Computing) with the packages tidyLPA and JMBayes2. Data analysis was conducted from June to August 2023.

## Results

### Study Population

Among the 1229 participants in the analysis, 533 (43.4%) were male; and the mean (SD) age was 59.5 (6.7) years. The baseline characteristics of participants stratified by weight loss are displayed in eTable 5 in Supplement 1. Across 4 years, 333 participants (27.1%) succeeded in attaining and maintaining a weight loss of at least 7%, which was observed in both groups (eFigure 3 in Supplement 1). After being stratified by PA trajectories, the 105 participants with sustained weight loss and high PA had higher education, less sedentary time, and lower total cholesterol levels than other participants (Table 1).

**Table 1.** Baseline Characteristics of Participants, Stratified by Weight Loss Categories and PA Trajectories

Characteristic	Participants, mean (SD)				*P* value
	Weight loss <7% (n = 896)		Weight loss ≥7% (n = 333)		
	Low PA (n = 663)	High PA (n = 233)	Low PA (n = 228)	High PA (n = 105)	
Age, y	59.4 (6.7)	58.5 (6.8)	60.8 (7.1)	59.3 (5.7)	.002
Sex, No. (%)					
Female	425 (64.1)	79 (33.9)	154 (67.5)	38 (36.2)	<.001
Male	238 (35.9)	154 (66.1)	74 (32.5)	67 (63.8)	
Race and ethnicity, No. (%)					
African American or non-Hispanic Black	124 (18.7)	32 (13.7)	45 (19.7)	7 (6.7)	.08
Hispanic	23 (3.5)	6 (2.6)	8 (3.5)	2 (1.9)	
White	499 (75.3)	186 (79.8)	169 (74.1)	91 (86.7)	
Other or multiracial^a^	17 (2.6)	9 (3.9)	6 (2.6)	5 (4.8)	
Education, No. (%)					
<13 y	112 (17.4)	27 (11.8)	37 (16.8)	16 (15.4)	.004
13-16 y	254 (39.4)	80 (34.9)	74 (33.6)	24 (23.1)	
>16 y	278 (43.2)	122 (53.3)	109 (49.5)	64 (61.5)	
Years since diabetes diagnosis	6.8 (6.9)	6.5 (5.7)	6.9 (6.5)	7.6 (6.5)	.57
Baseline body weight, kg	101.6 (19.0)	100.7 (18.2)	102.8 (18.7)	104.7 (19.9)	.26
Body weight at year 4, kg	102.2 (20.0)	100.5 (18.5)	89.8 (16.4)	90.7 (17.7)	<.001
BMI	36.3 (6.0)	34.0 (5.0)	37.3 (6.4)	35.7 (6.1)	<.001
Waist circumference, cm	114.7 (14.4)	112.6 (14.3)	115.2 (13.5)	116.2 (13.1)	.10
Smoking status, No. (%)					
Never	333 (50.2)	94 (40.3)	120 (52.6)	45 (42.9)	.009
Past	305 (46.0)	130 (55.8)	93 (40.8)	58 (55.2)	
Present	25 (3.8)	9 (3.9)	15 (6.6)	2 (1.9)	
Alcoholic drinks/wk	8.2 (21.8)	16.7 (32.0)	5.8 (13.0)	8.2 (16.8)	<.001
Sedentary h/wk	91.0 (13.3)	87.1 (12.0)	91.4 (13.0)	86.6 (12.8)	<.001
SBP, mm Hg	129.9 (17.0)	128.7 (16.0)	132.3 (18.1)	130.0 (17.2)	.14
DBP, mm Hg	70.2 (9.6)	71.8 (8.7)	70.1 (9.7)	72.1 (9.0)	.04
FPG, mg/dL	152.0 (44.0)	148.7 (42.5)	148.9 (42.1)	150.6 (44.6)	.68
HbA_1c_, %	7.3 (1.1)	7.0 (1.0)	7.2 (1.1)	7.1 (1.0)	.03
Total cholesterol, mg/dL	192.7 (38.0)	186.4 (35.9)	190.3 (36.7)	182.5 (33.7)	.02
Triglycerides, mg/dL	189.6 (126.0)	182.4 (129.9)	169.2 (97.2)	169.3 (97.1)	.09
LDL-c, mg/dL	112.4 (32.6)	110.3 (31.1)	111.5 (32.6)	106.6 (28.3)	34
HDL-c, mg/dL	43.5 (11.9)	41.1 (10.7)	45.2 (12.3)	42.5 (11.3)	.002
VLDL-c, mg/dL	36.8 (21.4)	35.0 (20.7)	33.5 (20.0)	33.4 (18.4)	.12
Serum creatinine, mg/dL	0.8 (0.2)	0.9 (0.2)	0.8 (0.2)	0.9 (0.2)	<.001
Insulin use, No. (%)	126 (19.0)	43 (18.5)	37 (16.2)	17 (16.2)	.76
Hypertension medications, No. (%)	503 (76.1)	169 (72.5)	174 (76.3)	73 (69.5)	.38
Medication for high cholesterol, No. (%)	365 (55.2)	134 (57.5)	123 (53.9)	57 (54.3)	.88
Aspirin used, No. (%)					
Every day	283 (42.7)	128 (54.9)	113 (49.6)	61 (58.1)	.007
Sometimes	305 (46.0)	80 (34.3)	95 (41.7)	36 (34.3)	
Never	75 (11.3)	25 (10.7)	20 (8.8)	8 (7.6)	
History of hypertension, No. (%)	563 (84.9)	191 (82.0)	201 (88.2)	87 (82.9)	.29
History of CVD, No. (%)	106 (16.0)	31 (13.3)	43 (18.9)	10 (9.5)	.12
Wear time, d	6.0 (0.6)	6.1 (0.5)	5.9 (0.6)	6.2 (0.5)	<.001
Intensive Lifestyle Intervention group, No. (%)	268 (40.4)	118 (50.6)	149 (65.4)	83 (79.0)	<.001

Abbreviations: BMI, body mass index (calculated as weight in kilograms divided by height in meters squared); CVD, cardiovascular disease; DBP, diastolic blood pressure; FPG, fasting plasma glucose; HbA_1c_, glycosylated hemoglobin; HDL-c, high-density lipoprotein cholesterol; LDL-c, low-density lipoprotein cholesterol; PA, physical activity; SBP, systolic blood pressure; VLDL-c, very-low-density lipoprotein cholesterol.

SI conversion factors: To convert creatinine to micromoles per liter, multiply by 88.4; FPG to millimoles per liter, multiply by 0.0555; HbA_1c_ to proportion of total hemoglobin, multiply by 0.01; HDL-c, LDL-c, total cholesterol, and VLDL-c to millimoles per liter, multiply by 0.0259; triglycerides to millimoles per liter, multiply by 0.0113.

^a^ Other race and ethnicity Asian, Pacific Islander, and other race and ethnicity.

### Weight Loss Stratified by PA Volume and Cardiovascular Events

During a median follow-up of 9.5 years, 54 participants with weight loss of 7% or greater (16.2%), 144 participants with weight loss of less than 7% (16.1%), 151 participants with low PA volume (16.9%), and 47 participants with high PA volume (13.9%) developed the primary outcome (eTable 6 in Supplement 1). No significant association was observed between weight loss or high PA volume with the primary outcome (eTable 6 and eFigure 4 in Supplement 1).

**Figure 1.**
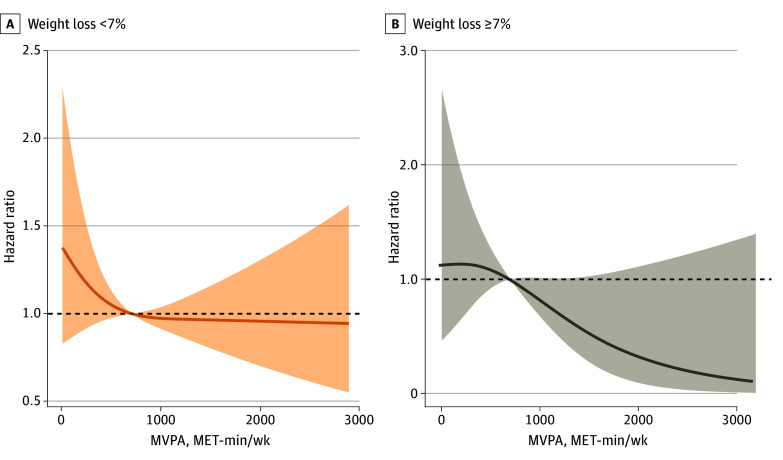
Associations Between 4-Year Accumulative Mean Physical Activity Volume With Primary Outcome, Stratified by Percentage of Weight Loss The primary outcome included cardiovascular death, nonfatal myocardial infarction or stroke, or hospitalization for angina. The association was estimated by the restricted cubic splines models. Shaded areas represent the 95% CI. The reference was set at 700 MET-min/wk. MVPA indicates moderate-to-vigorous physical activity.

The joint association between weight loss and PA volume with the risk of cardiovascular events was further assessed. Compared with the participants with low PA and no weight loss (reference group), individuals with high PA and weight loss had a significantly lower risk of the primary outcome (hazard ratio [HR], 0.39; 95% CI, 0.19-0.81) (Table 2; eFigure 5 in Supplement 1). The cardiovascular benefits from high PA volume were more significant among participants with weight loss of 7% or greater (*P* for interaction = .01). The association between 4-year accumulative PA volume and primary and secondary outcomes differed between those with and without weight loss of 7% or greater (Figure 1; eFigure 6 in Supplement 1). Higher PA volume was linearly associated with a decreased risk of cardiovascular events among participants with weight loss (1000 MET-min/wk: HR, 0.83; 95% CI, 0.68-1.01; 2000 MET-min/wk: HR, 0.33; 95% CI, 0.10-1.11). However, for those who failed to lose weight, the cardiovascular benefits appeared to reach a plateau at the guideline-recommended PA volume, at approximately 1000 MET-min/week (1000 MET-min/week: HR, 0.97; 95% CI, 0.91-1.04; 2000 MET-min/week: HR, 0.96; 95% CI, 0.70-1.31).

**Table 2.** Risk of Primary and Secondary Outcomes Stratified by Weight Loss and PA Trajectories

Group	Participants, No. (%)	Model 1, HR (95% CI)^a^	*P *value	Model 2, HR (95% CI)^b^	*P *value	*P *for interaction
**Primary outcome: CV death, nonfatal MI or stroke, or hospitalization for angina**						
Low PA and no weight loss	105 (15.8)	1 [Reference]	NA	1 [Reference]	NA	.01
Only high PA	39 (16.7)	0.87 (0.59-1.26)	.45	1.04 (0.70-1.53)	.86	
Only weight loss	46 (20.2)	1.29 (0.91-1.83)	.15	1.13 (0.78-1.62)	.52	
High PA and weight loss	8 (7.6)	0.36 (0.17-0.74)	.005	0.39 (0.19-0.81)	.01	
**Secondary outcome 1: CV death, nonfatal MI or stroke**						
Low PA and no weight loss	68 (10.3)	1 [Reference]	NA	1 [Reference]	NA	.10
Only high PA	22 (9.4)	0.76 (0.47-1.25)	.28	0.96 (0.57-1.59)	.86	
Only weight loss	34 (14.9)	1.46 (0.96-2.21)	.07	1.40 (0.91-2.15)	.13	
High PA and weight loss	6 (5.7)	0.45 (0.19-1.04)	.06	0.57 (0.24-1.36)	.21	
**Secondary outcome 2: all-cause death, nonfatal MI or stroke, or hospitalization for angina**						
Low PA and no weight loss	121 (18.3)	1 [Reference]	NA	1 [Reference]	NA	.002
Only high PA	43 (18.5)	0.83 (0.59-1.19)	.32	0.98 (0.68-1.42)	.92	
Only weight loss	61 (26.6)	1.48 (1.09-2.02)	.01	1.33 (0.96-1.84)	.08	
High PA and weight loss	9 (8.6)	0.35 (0.18-0.69)	.002	0.38 (0.19-0.75)	.006	
**Secondary outcome 3: all-cause death, nonfatal MI or stroke, hospitalization for angina, CABG, PCI, hospitalization for heart failure, carotid endarterectomy, or peripheral vascular disease**						
Low PA and no weight loss	154 (23.2)	1 [Reference]	NA	1 [Reference]	NA	.002
Only high PA	52 (22.3)	0.79 (0.57-1.09)	.15	0.88 (0.63-1.22)	.44	
Only weight loss	68 (29.8)	1.27 (0.95-1.70)	.10	1.14 (0.84-1.53)	.40	
High PA and weight loss	10 (9.5)	0.29 (0.15-0.56)	<.001	0.31 (0.16-0.60)	<.001	

Abbreviations: CABG, coronary artery bypass grafting; CV, cardiovascular; MI, myocardial infarction; NA, not applicable; PA, physical activity; PCI, percutaneous coronary intervention.

^a^ Model 1 was adjusted for age, race and ethnicity, and sex.

^b^ Model 2 was adjusted for age, race and ethnicity, sex, history of cardiovascular disease, fasting plasma glucose level, history of hypertension, insulin use, aspirin use, sedentary time, drinking status, smoking status, triglyceride level, and treatment group.

### Joint Model of Weight Loss, Longitudinal PA, and the Primary Outcome

**Figure 2.**
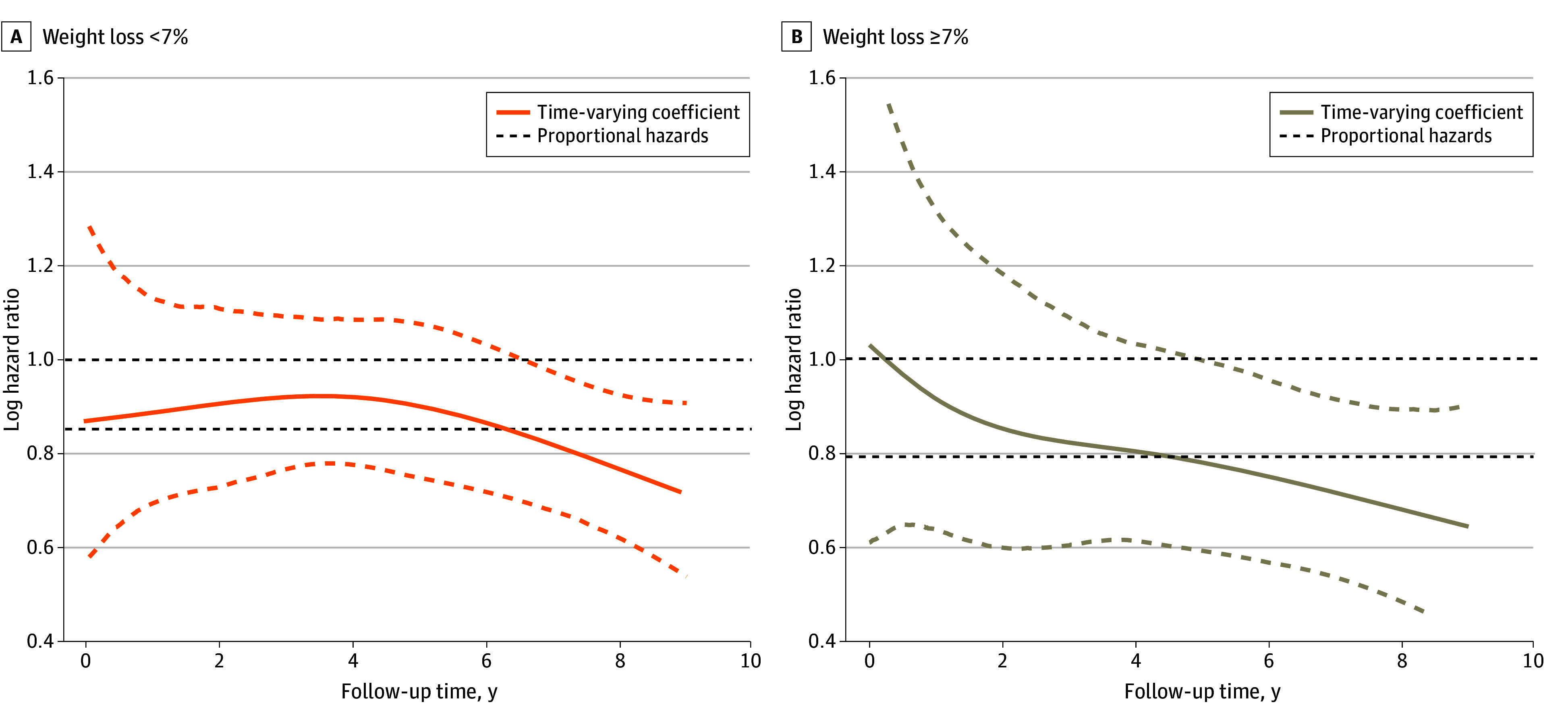
Time-Varying Associations Between Physical Activity Volume and the Primary Outcome, Stratified by Percentage of Weight Loss The primary outcome included cardiovascular death, nonfatal myocardial infarction or stroke, or hospitalization for angina. The association was estimated by the natural spline models. The orange and tan dashed lines indicate the 95% CIs.

Per unit of longitudinal PA increase was associated with a reduction in cardiovascular events (HR, 0.84; 95% CI, 0.75-0.93) (Table 3). The reduction of risk of the primary outcome was associated with the current value of PA volume; in the model with weight loss alone, the result was not statistically significant (HR, 0.86; 95% CI, 0.56-1.33). The benefits from a steeper PA increase may differ from the weight loss categories (slope of joint model: *P* for interaction = .02). During weight loss, the cardiovascular benefits from increasing PA differed over time, and the model achieved statistical significance around the fourth year (*P* for interaction = .07) (Table 3 and Figure 2B). Notably, among those without weight loss, the benefits from increasing PA remained constant over time (2 years: HR, 0.60; 95% CI, 0.84-1.16; 4 years: HR, 0.61; 95% CI, 0.80-1.03; 6 years: HR, 0.58; 95% CI, 0.76-0.96), while among those who did not lose weight, the results were not statistically significant at 2, 4, or 6 years (2 years: HR, 0.73; 95% CI, 0.90-1.12; 4 years: HR, 0.77; 95% CI, 0.92-1.08; 6 years: HR, 0.73; 95% CI, 0.86-1.02) (Figure 2A).

**Table 3.** Multivariable JMs for Longitudinal Evaluation of Logarithmic Transformation of PA + 1 and the Primary Outcome^a^

Model and variables	HR (95% CI)	*P* value	*P* value for interaction	R hat
JM (basic)				
Weight loss	0.86 (0.56-1.33)	.52	NA	1.00
Log (PA + 1)	0.84 (0.75-0.93)	<.001	NA	1.01
JM (value)^b^				
Weight loss	1.20 (0.51-2.58)	.66	.39	1.05
Log (PA + 1)	0.86 (0.76-0.96)	.01		1.05
JM (slope)^b^				
Weight loss	0.42 (0.09-1.25)	.12	.02	1.12
Log (PA + 1)	0.12 (0-5.27)	.23		1.31
JM (time, linear)^c^				
Weight loss	0.89 (0.59-1.35)	.58	.07	1.00
Log (PA + 1)	0.96 (0.80-1.15)	.73		1.02
JM (time, ns)^c^				
Weight loss	0.88 (0.57-1.34)	.57	NA	1.01
Log (PA + 1)	0.98 (0.73-1.35)	.90	NA	1.47
Log (PA + 1), 0	0.96 (0.66-1.35)	NA	.90	1.31
Log (PA + 1), 1	0.71 (0.40-1.25)	NA	.26	1.36
Log (PA + 1), 4	0.76 (0.58-1.03)	NA	.07	1.12

Abbreviations: HR, hazard ratio; JM, joint model; NA, not applicable; ns, natural cubic splines; PA, physical activity.

^a^ Participants who completed baseline PA with both PA measurements at year 1 and 4 were included (n = 1229), and 198 events were observed. Time-varying effects were conducted using the linear mixed-effects and natural cubic spline models.

^b^ *P* for interaction was calculated by adding multiplicative interaction terms of weight loss and log (PA + 1).

^c^ *P* for interaction was calculated by adding multiplicative interaction terms of time and log (PA + 1).

### Sensitivity and Subgroup Analysis

The associations between high PA volume, weight loss, and risk of cardiovascular events were still significant when adjusting the weight loss threshold to that consistent with the ADA guideline recommendations (eTable 7 in Supplement 1), using a more relaxed threshold of moderate-to-vigorous PA duration (eTable 8 in Supplement 1), involving the cluster model with a lower BIC but smaller sample size (eTable 9 in Supplement 1), excluding 78 participants who experienced the primary outcome in the first 4 years (eTable 10 in Supplement 1), and treating weight loss as a continuous variable (eTable 11 in Supplement 1). Similar results were found when including participants with at least 1 instance of follow-up PA data in year 1 and year 4 (eTable 12 and eFigure 7 in Supplement 1). After stratifying by treatment group, sex, age, and race, consistent results were observed in the subgroup analysis, and no further interaction effects were observed (eFigure 8 in Supplement 1).

## Discussion

This post hoc analysis of the Look AHEAD trial was performed in individuals with T2D and overweight or obesity to determine the joint association of weight loss and PA with the risk of cardiovascular events. This study found that only weight loss or only increased PA were not associated with a lower risk of cardiovascular events. However, maintaining weight loss combined with a higher moderate-to-vigorous PA volume was associated with cardiovascular benefits. During and after the weight loss period, the cardiovascular benefits from PA may be enhanced. These findings highlight the contribution of increasing PA to reduce the risk of cardiovascular events when implementing a weight loss program in those with T2D and overweight or obesity.

Our findings are supported by the previous studies that investigated the benefits of weight loss and/or increased PA in individuals with T2D and overweight and obesity.^16,27,28^ Studies on single lifestyle changes, ie, diet-induced weight loss only or increasing PA only, have identified their benefits for improving insulin sensitivity and glycemic control in patients with overweight and obesity or T2D.^16,27,28^ The Look AHEAD trial further investigated the cardiovascular effects by providing weight loss–oriented lifestyle management in individuals with T2D and overweight or obesity but found a nonsignificant difference between the ILI and DSE groups.^14^ Several post hoc analyses were conducted and demonstrated achieving greater weight loss magnitudes, maintaining weight loss, and increasing PA volumes were associated with a lower risk of cardiovascular events.^8,10,11^ Of note, these clinical trials did not consider the joint association of weight loss and increasing PA. Compared with weight loss only or exercise only, prior studies reported a greater visceral fat reduction and insulin sensitivity improvement when incorporating both.^18,29^ However, the additive effect was not statistically significant in other studies,^16,17^ and the risks of developing T2D were at similar levels when losing weight regardless of PA.^30^

In the current study, increasing PA only or losing weight only were not associated with cardiovascular benefits in adults with T2D and overweight or obesity. Consistent with prior post hoc analyses, losing 7% or greater of body weight may be insufficient for the individuals in Look AHEAD, who had a very high baseline body mass index (BMI; mean [SD], 36.4 [6.0]), and the cardiovascular benefits from increasing PA may be modest in such population.^8,11^ Combined with the previous and conflicting findings of incorporating weight loss and exercise, it suggests that the benefits from PA may vary among individuals with different baseline characteristics.^11,18,29^ This may also partially explain the lower risk of cardiovascular events we observed in those incorporating weight loss and high PA volume and the enhanced benefits from increasing PA during and after losing weight. Nevertheless, our findings should be interpreted cautiously because there was limited evidence on the complicated associations between increasing PA, lifestyle-induced weight loss, cardiovascular benefits, and their joint associations. Based on the recent studies, our findings imply the cardiovascular benefits of moderate-to-vigorous PA may vary among individuals with differing characteristics.^8,17,18^

The cardiovascular benefits attained from increasing PA and weight loss are multifaceted.^17,31^ Prior systematic reviews^31,32^ summarized the mechanisms, including improved metabolism and vascular health as well as stimulating the release of cardiovascular-protective exerkines. However, the mechanisms that weight loss enhanced the cardiovascular benefits of PA in individuals with T2D and overweight or obesity were understudied. This population may have greater ectopic fat deposition and muscle atrophy, which may impair their already weakened skeletal muscle biology due to lipotoxic effects.^33^ However, many exercise-induced cardiovascular benefits are driven by skeletal muscle biology.^17,31^ Lipotoxic effects may be mitigated through weight loss or exercise, and exercise may additionally improve the energy metabolisms and mitochondrial functions within the skeletal muscles and liver.^17,34,35^ These physiological responses (eg, insulin clearance and circulating proteins related to skeletal muscle energy metabolism and mitochondrial biology) may also be enhanced by the reduction of ectopic fat through weight loss.^17,34^ Thus, weight loss may provide positive feedback for the benefits of increasing PA, and incorporating both may reverse the pathological changes of T2D and obesity.^17,33^

This study highlights the association between combined weight loss and increased PA with a reduced risk of cardiovascular events in individuals with T2D and overweight or obesity. First, maintaining a high PA volume and losing weight are important, but the combination may be more beneficial in this population. It may be more helpful when clinicians give prescriptions and lifestyle suggestions considering the association between cardiovascular benefits, PA, and weight loss with the individual’s characteristics. Second, future RCTs targeting individuals with T2D and overweight or obesity should consider lifestyle interventions based on basic characteristics or combining weight loss and increased PA to elucidate the causal relationships between exercise, weight loss, and cardiovascular events. Third, this study provides new evidence to support the current guideline recommendations on maintaining weight loss and increasing PA volume to lower risk of cardiovascular events.^3,5,6,7^

### Strengths and Limitations

The present study had several strengths, including characterizing long-term PA patterns using repeated objective measures from accelerometry in individuals with T2D and overweight or obesity and using data from a multicenter RCT with a considerable sample size and nearly 10-year follow-up period. The study also encompassed predetermined primary and secondary cardiovascular outcomes.

However, this study also had several limitations. First, as a post hoc analysis of an RCT involving a cohort design and collapsing the randomization groups, there may be some unmeasured mixed responses. We have addressed this by adjusting for randomized assignment as a covariate and conducting a subgroup analysis stratified by the treatment group. Second, the study included the individuals in the PA substudy, excluding many due to missing PA data in follow-up, which may be related to failure in weight loss at those time points. Nonetheless, the joint models involved in the analyses allowed for more comprehensive use of longitudinal PA data, thereby mitigating the impact of missing data. Third, as previously mentioned, establishing a clear causal relationship between PA, weight loss, and cardiovascular outcomes remains challenging due to the complexity of their interactions and time-varying effects, which are difficult to fully capture by the current cohort design and statistical models. Despite this, the robustness of our primary findings was enhanced by using various statistical models, trajectory analyses, sensitivity and subgroup analyses, and corroborating with prior studies.^17,18^ Moreover, similar to other observational studies, the influence of the unmeasured confounders could not be excluded, but we have excluded the effects of the key confounders.

## Conclusions

Among individuals with T2D and overweight or obesity, combined weight loss and maintaining high PA volume were associated with reduced risks of adverse cardiovascular outcomes. The cardiovascular benefits of increased PA appeared to vary over the weight loss period. Weight loss may enhance the benefits from PA, and incorporating both might help in lowering the risk of cardiovascular events further.

## Notes

Supplement 1.
eMethods.
**eTable 1.** Baseline Characteristics of Included and Excluded Participants **eTable 2.** Moderate-to-Vigorous Physical Activity Group-Based Trajectory Model Fit Summary and Sample Sizes **eTable 3.** PA Volumes at Examination Visits by Moderate-to-Vigorous Physical Activity Trajectory Groups **eTable 4.** Results of Variance Inflation Factor in the Fully Adjusted Model for Primary Outcome **eTable 5.** Baseline Characteristics of Participants Stratified by Weight Loss **eTable 6.** Risk of Primary and Secondary Outcomes Stratified by Only Weight Loss Categories or PA Trajectory **eTable 7.** Risk of Primary and Secondary Outcomes Stratified by Weight Loss and MVPA Trajectories Using the Criteria of the American Diabetes Association **eTable 8.** Risk of Primary and Secondary Outcomes Stratified by Weight Loss and MVPA Trajectories Using MVPA Accumulated in 1-Minute Bouts **eTable 9.** Risk of Primary and Secondary Outcomes Stratified by Weight Loss and MVPA Volume Trajectories by Model 6 **eTable 10.** Risk of Primary and Secondary Outcomes Stratified by Weight Loss and MVPA Volume Trajectories Excluding the Participants Experienced Outcomes Within the First 4 Years **eTable 11.** Risk of Primary and Secondary Outcomes Stratified by MVPA Volume Trajectories and Treated Weight Loss as a Continuous Variable **eTable 12.** Multivariable Joint Models for Longitudinal Evaluation of Logarithmic Transformation of (PA + 1) and Primary Outcome Including Participants With Baseline and at Least 1 Follow-Up PA Measures **eFigure 1.** Flowchart for Participant Selection **eFigure 2.** Moderate-to-Vigorous Physical Activity Volume Trajectory Model Establishment **eFigure 3.** Sankey Diagram of 4-Year Body Weight Stratified by Treatment Groups **eFigure 4.** Cumulative Incidence Estimates for the Primary Outcome and 3 Secondary Outcomes Among Participants Stratified by Weight Loss Categories **eFigure 5.** Cumulative Incidence Estimates for the Primary and Secondary Outcomes Stratified by Weight Loss Categories and PA Trajectories **eFigure 6.** Associations Between 4-Year Accumulative Mean PA Volume With Secondary Outcomes Stratified by Weight Loss Categories **eFigure 7.** Time-Varying Associations Between PA Volume and Primary Outcomes Stratified by Weight Loss Including Participants With at Least 1 Follow-Up PA Data **eFigure 8.** Subgroup Analysis of the Risk of Primary Outcome
eReferences.

Supplement 2.
Data Sharing Statement
