# Supplementary material for: Physical Activity and Weight Loss Among Adults With Type 2 Diabetes and Overweight or Obesity: A Post Hoc Analysis of the Look AHEAD Trial
Source: JAMA Netw Open. 2024 Feb 22;7(2):e240219. doi: 10.1001/jamanetworkopen.2024.0219 (PMC10884882; doi:10.1001/jamanetworkopen.2024.0219)
Supplement: Supplement 1. — eMethods. eTable 1. Baseline Characteristics of Included and Excluded Participants eTable 2. Moderate-to-Vigorous Physical Activity Group-Based Trajectory Model Fit Summary and Sample Sizes eTable 3. PA Volumes at Examination Visits by Moderate-to-Vigorous Physical Activity Trajectory Groups eTable 4. Results of Variance Inflation Factor in the Fully Adjusted Model for Primary Outcome eTable 5. Baseline Characteristics of Participants Stratified by Weight Loss eTable 6. Risk of Primary and Secondary Outcomes Stratified by Only Weight Loss Categories or PA Trajectory eTable 7. Risk of Primary and Secondary Outcomes Stratified by Weight Loss and MVPA Trajectories Using the Criteria of the American Diabetes Association eTable 8. Risk of Primary and Secondary Outcomes Stratified by Weight Loss and MVPA Trajectories Using MVPA Accumulated in 1-Minute Bouts eTable 9. Risk of Primary and Secondary Outcomes Stratified by Weight Loss and MVPA Volume Trajectories by Model 6 eTable 10. Risk of Primary and Secondary Outcomes Stratified by Weight Loss and MVPA Volume Trajectories Excluding the Participants Experienced Outcomes Within the First 4 Years eTable 11. Risk of Primary and Secondary Outcomes Stratified by MVPA Volume Trajectories and Treated Weight Loss as a Continuous Variable eTable 12. Multivariable Joint Models for Longitudinal Evaluation of Logarithmic Transformation of (PA + 1) and Primary Outcome Including Participants With Baseline and at Least 1 Follow-Up PA Measures eFigure 1. Flowchart for Participant Selection eFigure 2. Moderate-to-Vigorous Physical Activity Volume Trajectory Model Establishment eFigure 3. Sankey Diagram of 4-Year Body Weight Stratified by Treatment Groups eFigure 4. Cumulative Incidence Estimates for the Primary Outcome and 3 Secondary Outcomes Among Participants Stratified by Weight Loss Categories eFigure 5. Cumulative Incidence Estimates for the Primary and Secondary Outcomes Stratified by Weight Loss Categories and PA Trajectories eFig [file jamanetwopen-e240219-s001.pdf]

Supplemental Online Content

Huang Z, Zhuang X, Huang R, et al. Physical activity and weight loss among adults with type 2 diabetes and overweight or obesity. *JAMA Netw Open.* 2024;7(2):e240219. doi:10.1001/jamanetworkopen.2024.0219

eMethods.

eTable 1. Baseline Characteristics of Included and Excluded Participants

eTable 2. Moderate-to-Vigorous Physical Activity Group-Based Trajectory Model Fit Summary and Sample Sizes

eTable 3. PA Volumes at Examination Visits by Moderate-to-Vigorous Physical Activity Trajectory Groups

eTable 4. Results of Variance Inflation Factor in the Fully Adjusted Model for Primary Outcome

eTable 5. Baseline Characteristics of Participants Stratified by Weight Loss

eTable 6. Risk of Primary and Secondary Outcomes Stratified by Only Weight Loss Categories or PA Trajectory

eTable 7. Risk of Primary and Secondary Outcomes Stratified by Weight Loss and MVPA Trajectories Using the Criteria of the American Diabetes Association

eTable 8. Risk of Primary and Secondary Outcomes Stratified by Weight Loss and MVPA Trajectories Using MVPA Accumulated in 1-Minute Bouts

eTable 9. Risk of Primary and Secondary Outcomes Stratified by Weight Loss and MVPA Volume Trajectories by Model 6

eTable 10. Risk of Primary and Secondary Outcomes Stratified by Weight Loss and MVPA Volume Trajectories Excluding the Participants Experienced Outcomes Within the First 4 Years

eTable 11. Risk of Primary and Secondary Outcomes Stratified by MVPA Volume Trajectories and Treated Weight Loss as a Continuous Variable

eTable 12. Multivariable Joint Models for Longitudinal Evaluation of Logarithmic Transformation of (PA + 1) and Primary Outcome Including Participants With Baseline and at Least 1 Follow-Up PA Measures

eFigure 1. Flowchart for Participant Selection

eFigure 2. Moderate-to-Vigorous Physical Activity Volume Trajectory Model Establishment

eFigure 3. Sankey Diagram of 4-Year Body Weight Stratified by Treatment Groups

eFigure 4. Cumulative Incidence Estimates for the Primary Outcome and 3 Secondary Outcomes Among Participants Stratified by Weight Loss Categories

eFigure 5. Cumulative Incidence Estimates for the Primary and Secondary Outcomes Stratified by Weight Loss Categories and PA Trajectories

eFigure 6. Associations Between 4-Year Accumulative Mean PA Volume With Secondary Outcomes Stratified by Weight Loss Categories

eFigure 7. Time-Varying Associations Between PA Volume and Primary Outcomes Stratified by Weight Loss Including Participants With at Least 1 Follow-Up PA Data

© 2024 Huang Z et al. *JAMA Network Open.*

eFigure 8. Subgroup Analysis of the Risk of Primary Outcome

eReferences.

This supplemental material has been provided by the authors to give readers additional information about their work.

eMethods.

Eligibility criteria

From Aug 22, 2001, to April 30, 2004, the Look AHEAD trial recruited the adults in all ethnic groups based on the data from the Third National Health and Nutrition Survey (NHANES III), diagnosed with type 2 diabetes mellitus, with overweight/obese adults (body mass index [BMI] ≥ 25 kg/m^2 or ≥ 27 kg/m^2 if taking insulin), aged 45 to 76 years, systolic blood pressure < 160 mmHg, diastolic blood pressure < 100 mmHg, triglycerides < 600 mg/dL, glycosylated hemoglobin ≤ 11% (97 mmol/mol from 16 clinical centers in the USA.

Exclusion Criteria

Exclusion criteria included individuals with type 1 diabetes, diseases limiting lifespan or affecting safety, and individuals who were unable to complete a maximal graded exercise test or 2 weeks of self-monitoring of diet and activity.

Study Interventions

Participants in the ILI arm aimed at achieving and maintaining a weight loss of ≥ 7% through individual approaches and group counselling during the first year. Specific interventions for weight loss included restricted caloric intakes to 1200 to 1800 kcal/day and increased non-supervised moderate-to-vigorous physical activity (PA) progressively to ≥ 175 minutes per week (min/week). Participants in the DSE arm were given educational group sessions three times per year each year for 4.0 to 6.5 years after study randomization. The intervention details have been previously published.[1,2]

© 2024 Huang Z et al. *JAMA Network Open.*

Adjustment Covariates

Demographic and clinical characteristics were assessed before randomization by standard protocols described previously.[1] Briefly, the age, race, sex, history of cardiovascular events, history of hypertension, insulin use, aspirin use, drinking status and smoking status were assessed by self-report questionnaires. Ethnicity groups included African American / Non-Hispanic Black, Hispanic, White and Other/Mixed. Other/Mixed, included Asian, Pacific Islander and others were collapsed because of the small sample size in this population. Blood draws for laboratory measurement (fasting plasma glucose and triglyceride) were conducted by the trained staff. Sedentary time, derived from the accelerometers, was defined as any physical activity < 1.5 metabolic equivalents of tasks (METs) accumulated for over 20-minute bouts.[3,4] A mean sedentary time of 3 visits (Years 0, 1 and 4) was calculated.

Comparison of Weight loss categories and PA trajectory respectively

In the Look AHEAD trial, participants were categorized according to the 4-year weight loss percentage < 7% (shown as no weight loss) and ≥ 7% (shown as weight loss). Simultaneously, participants were clustered into two PA trajectory group, low and high PA volume. The Kaplan–Meier method and the log-rank test were used to estimate and compare the cumulative incidence of each outcome by weight loss categories and PA trajectory groups respectively. Cox proportional hazard regression models were used to estimate hazard ratios (HR) and 95% confidence interval (CI) for the association between each outcome and weight loss categories and PA trajectory groups respectively. Two multivariable models were built to adjust the potential confounders. Model 1 was adjusted for age, sex and race at baseline. Model 2 was additionally adjusted for history of cardiovascular events, fasting plasma glucose, history of hypertension, insulin use, aspirin use, sedentary time, drinking status, smoking status,

© 2024 Huang Z et al. *JAMA Network Open.*

triglyceride and treatment arm at baseline.

Explanation of PA trajectory model selection

The selection of the PA trajectory model was guided by several criteria: a lower Akaike Information Criterion (AIC) and Bayesian Information Criterion (BIC), a higher mean posterior probability (>0.7), and a sufficient number of observations in each group.[5,6] Clustering PA trajectories using models 2 and 6 into either 2 or 3 groups met the first two criteria, as shown in eTable 2 and eFigure 2A. Given our study's focus on the joint association of PA and weight loss, each stratified group within the PA trajectory and weight loss needed to contain at least 5% of the total participants to ensure adequate sample size. In this context, clustering by models 2 and 6 into 2 groups was preferable (eTable 2). Both models yielded similar BIC and AIC values, but model 2 was chosen for the main analysis due to its larger sample size in the weight loss and high PA volume group, a challenging achievement for individuals with type 2 diabetes and overweight/obesity.[7,8] To validate the robustness of our approach, clustering by model 6 into 2 groups was also conducted as a sensitivity analysis.

Explanation of joint models

In our study, we employed joint modelling to concurrently assess the associations among PA, weight loss, time, and cardiovascular outcomes. The longitudinal relationship between PA, weight loss, and time was analysed using a linear mixed-effects model with random intercepts and slopes. Aligning with our study objectives and the temporal relationship between weight loss and PA, weight loss was categorized as either <7% or ≥7% over 4 years. The basic joint model integrated this linear mixed-effect model with a Cox model, adjusting for previously mentioned covariates. This model also allowed us to estimate the current level and slope within

© 2024 Huang Z et al. *JAMA Network Open.*

the mixed-effect model.[9] We further refined these estimates by introducing a multiplicative interaction term to assess the combined effect of PA and weight loss.

To explore time-varying effects, we examined both linear and nonlinear associations, as well as interactions between PA and time. For the linear association, we included a multiplicative interaction term of PA and time. In the nonlinear analysis, we utilized a natural spline with two knots placed at 1 and 4 years, combined with a B spline restricted to the range of 0 to 9.5 years. This approach was chosen because, in Look AHEAD, significant weight changes were observed in the first year of intervention, followed by weight regain, and a decrease in visit and intervention frequency after 4-year interventions.[2] This period's complexity warranted a more nuanced analysis of the nonlinear time effects. However, due to PA measurements being limited to baseline, year 1, and year 4, fitting a more complex nonlinear model between PA and time was challenging.[1] The parameters of the B splines were aligned with the median follow-up duration. Additionally, our joint model accommodated missing values in the longitudinal process. Therefore, for sensitivity analysis, we included participants who had PA measurements at baseline and at least one follow-up at year 1 or year 4 (eTable 12).

eTable 1. Baseline Characteristics of Included and Excluded Participants

	Overall (n=2570)	Excluded (n=1341)	Included (n=1229)	*P*
Age, Years	59.0 (6.9)	58.6 (7.0)	59.5 (6.7)	0.002
Male (%)	1109 (43.2)	576 (43.0)	533 (43.4)	0.86
Race (%)				<0.001
African American / Non-Hispanic Black	505 (19.6)	297 (22.1)	208 (16.9)	
Hispanic	129 (5.0)	90 (6.7)	39 (3.2)	
White	1837 (71.5)	892 (66.5)	945 (76.9)	
Other/Mixed	99 (3.9)	62 (4.6)	37 (3.0)	
Education (%)				0.01
< 13 years	415 (16.5)	223 (16.9)	192 (16.0)	
13 - 16 years	971 (38.6)	539 (41.0)	432 (36.1)	
> 16 years	1127 (44.8)	554 (42.1)	573 (47.9)	
Years of DM diagnosed	6.8 (6.4)	6.7 (6.3)	6.9 (6.6)	0.65
Baseline body weight (kg)	103.1 (19.3)	104.1 (19.7)	101.9 (18.9)	0.003
Body weight at Year 4 (kg)	99.5 (19.6)	100.5 (19.6)	98.6 (19.6)	0.02
BMI (kg/m^2)	36.4 (6.0)	36.8 (6.0)	36.0 (6.0)	0.002
Waist girth (cm)	115.5 (14.6)	116.3 (14.9)	114.5 (14.1)	0.001
Smoking status (%)				0.65
Never	1258 (49.1)	666 (49.9)	592 (48.2)	
Past	1203 (46.9)	617 (46.3)	586 (47.7)	
Present	102 (4.0)	51 (3.8)	51 (4.1)	
Alcoholic used (drinks/week)	8.5 (21.1)	7.7 (19.4)	9.3 (22.8)	0.05
Sedentary (hours/week)	85.9 (17.9)	82.1 (20.6)	90.0 (13.1)	<0.001
SBP (mmHg)	131.2 (17.1)	132.2 (17.0)	130.1 (17.1)	0.002
DBP (mmHg)	71.2 (9.5)	71.6 (9.5)	70.6 (9.4)	0.007
FPG (mg/dL)	151.4 (44.8)	152.0 (46.1)	150.7 (43.4)	0.46
HbA1c (%)	7.2 (1.1)	7.3 (1.2)	7.2 (1.1)	0.04
Total cholesterol (mg/dL)	191.1 (38.0)	192.0 (38.8)	190.2 (37.1)	0.23
Triglycerides (mg/dL)	180.4 (117.7)	178.2 (115.6)	182.7 (119.8)	0.33
LDL-c (mg/dL)	112.7 (32.6)	114.0 (33.1)	111.4 (32.0)	0.04
HDL-c (mg/dL)	43.3 (12.0)	43.4 (12.3)	43.3 (11.8)	0.79
VLDL-c (mg/dL)	35.1 (20.1)	34.6 (19.5)	35.6 (20.8)	0.21

© 2024 Huang Z et al. *JAMA Network Open.*

Serum Creatinine (mg/dL)	0.8 (0.2)	0.8 (0.2)	0.8 (0.2)	0.99
Insulin use (%)	473 (18.4)	250 (18.6)	223 (18.1)	0.78
Hypertension medications (%)	1934 (75.3)	1015 (75.7)	919 (74.9)	0.65
Medication for High cholesterol (%)	1381 (53.8)	702 (52.4)	679 (55.3)	0.15
Aspirin used (%)				0.14
Every day	1167 (45.6)	582 (43.8)	585 (47.6)	
Sometimes	1122 (43.8)	606 (45.6)	516 (42.0)	
Never	270 (10.6)	142 (10.7)	128 (10.4)	
History of Hypertension (%)	2204 (85.8)	1162 (86.7)	1042 (84.8)	0.20
History of CVD (%)	384 (14.9)	194 (14.5)	190 (15.5)	0.52
Intensive Lifestyle Intervention (%)	1286 (50.0)	668 (49.8)	618 (50.3)	0.84

Data are n (%) or mean (SD). PA: physical activity; DM, diabetes mellitus; BMI, body mass index; SBP, systolic blood pressure; DBP, diastolic blood pressure; FPG, fasting plasma glucose; HbA1c, glycosylated haemoglobin; LDL-c, low-density lipoprotein cholesterol; HDL-c, high-density lipoprotein cholesterol; VLDL-c, very-low-density lipoprotein cholesterol; CVD, cardiovascular disease. Other ethnicities include American Indians and Asian Americans.

© 2024 Huang Z et al. *JAMA Network Open.*

eTable 2. Moderate-to-Vigorous Physical Activity Group-Based Trajectory Model Fit Summary and Sample Sizes (n=1,229)

Model	AIC	BIC	Mean posterior probability	Stratified Sample size (No WL)	Stratified Sample size (WL)
Class = 2					
1	62933	62984	1.00/0.93	844/52	301/32
**2**	**57066**	**57133**	**0.98/0.96**	**663/233**	**228/105**
3	62909	62975	1.00/0.94	844/52	301/32
4/5	NA	NA	NA	NA	NA
6	56874	56971.45	0.98/0.96	704/192	241/92
Class = 3					
1	62941	63013	NA/0.51/0.90	NA/841/55	NA/300/33
2	54985	55087	0.96/0.96/0.96	320/504/72	180/177/48
3	62917	63004	NA/0.51/0.92	NA/841/55	NA/300/33
4/5	NA	NA	NA	NA	NA
6	54905	55053	0.96/0.97/0.96	351/495/50	117/183/33

AIC, Akaike's information criterion; BIC, Bayesian information criterion; WL, weight loss.
Model 1: Equal variances, and covariances fixed to 0.
Model 2: Varying variances and covariances fixed to 0.
Model 3: Equal variances and equal covariances.
Model 4 & 5 is not available.
Model 6: Varying variances and varying covariances.

© 2024 Huang Z et al. *JAMA Network Open*.

eTable 3. PA Volumes at Examination Visits by Moderate-to-Vigorous Physical Activity Trajectory Groups, Median [IQR]

Group	Baseline	Year 1	Year 4
Low physical activity (n=891)	112 [0-348]	127 [0-405]	49.5 [0-227]
High physical activity (n=338)	891 [303-1542]	1315 [625-2125]	909 [334-1531]

eTable 4. Results of Variance Inflation Factor in the Fully Adjusted Model for Primary Outcome

Variables	df	Variance inflation factor	
		GVIF	VIF
Age	1	1.32	1.15
Race	3	1.25	1.04
Sex	1	1.35	1.16
History of CV events	1	1.27	1.13
Fasting plasma glucose	1	1.11	1.06
History of hypertension	1	1.06	1.03
Insulin used	1	1.06	1.03
Sedentary time	1	1.28	1.13
Aspirin used	2	1.27	1.06
Drinking habits	1	1.14	1.07
Smoking history	2	1.24	1.06
Triglycerides	1	1.17	1.08
Treatment arm	1	1.10	1.05
GLOBAL	20	-	-

CV, cardiovascular; GVIF, generalized variance inflation factor; df, degree of freedom.

eTable 5. Baseline Characteristics of Participants Stratified by Weight Loss

	**Overall (n=1229)**	**Weight loss < 7% (n=896)**	**Weight loss ≥ 7% (n=333)**	***P***
Age, Years	59.5 (6.7)	59.1 (6.7)	60.3 (6.7)	0.006
Male (%)	533 (43.4)	392 (43.8)	141 (42.3)	0.71
Race (%)				0.87
African American / Non-Hispanic Black	208 (16.9)	156 (17.4)	52 (15.6)	
Hispanic	39 (3.2)	29 (3.2)	10 (3.0)	
White	945 (76.9)	685 (76.5)	260 (78.1)	
Other/Mixed	37 (3.0)	26 (2.9)	11 (3.3)	
Education (%)				0.03
< 13 years	192 (16.0)	139 (15.9)	53 (16.4)	
13 - 16 years	432 (36.1)	334 (38.3)	98 (30.2)	
> 16 years	573 (47.9)	400 (45.8)	173 (53.4)	
Years of DM diagnosed	6.9 (6.6)	6.8 (6.6)	7.1 (6.5)	0.37
Baseline body weight (kg)	101.9 (18.9)	101.3 (18.8)	103.4 (19.0)	0.09
Body weight at Year 4 (kg)	98.6 (19.6)	101.7 (19.6)	90.1 (16.8)	<0.001
BMI (kg/m^2)	36.0 (6.0)	35.7 (5.8)	36.8 (6.3)	0.006
Waist girth (cm)	114.5 (14.1)	114.1 (14.4)	115.5 (13.4)	0.13
Smoking status (%)				0.43
Never	592 (48.2)	427 (47.7)	165 (49.5)	
Past	586 (47.7)	435 (48.5)	151 (45.3)	
Present	51 (4.1)	34 (3.8)	17 (5.1)	
Alcoholic used (drinks/week)	9.3 (22.8)	10.4 (25.1)	6.6 (14.4)	0.009
Sedentary (hours/week)	59.8 (9.5)	59.8 (9.6)	59.8 (9.1)	0.98
SBP (mmHg)	130.1 (17.1)	129.6 (16.8)	131.6 (17.8)	0.07
DBP (mmHg)	70.6 (9.4)	70.6 (9.4)	70.7 (9.5)	0.87
FPG (mg/dL)	150.7 (43.4)	151.2 (43.6)	149.4 (42.8)	0.53
HbA1c (%)	7.2 (1.1)	7.2 (1.1)	7.2 (1.1)	0.59
Total cholesterol (mg/dL)	190.2 (37.1)	191.1 (37.5)	187.8 (35.9)	0.17
Triglycerides (mg/dL)	182.7 (119.8)	187.8 (127.0)	169.3 (97.0)	0.02
LDL-c (mg/dL)	111.4 (32.0)	111.9 (32.2)	110.0 (31.3)	0.35
HDL-c (mg/dL)	43.3 (11.8)	42.9 (11.6)	44.3 (12.1)	0.05
VLDL-c (mg/dL)	35.6 (20.8)	36.3 (21.2)	33.5 (19.5)	0.03

© 2024 Huang Z et al. *JAMA Network Open.*

Serum Creatinine (mg/dL)	0.8 (0.2)	0.8 (0.2)	0.8 (0.2)	0.39
Insulin use (%)	223 (18.1)	169 (18.9)	54 (16.2)	0.32
Hypertension medications (%)	919 (74.9)	672 (75.2)	247 (74.2)	0.78
Medication for High cholesterol (%)	679 (55.3)	499 (55.8)	180 (54.1)	0.63
Aspirin used (%)				0.10
Every day	585 (47.6)	411 (45.9)	174 (52.3)	
Sometimes	516 (42.0)	385 (43.0)	131 (39.3)	
Never	128 (10.4)	100 (11.2)	28 (8.4)	
History of Hypertension (%)	1042 (84.8)	754 (84.2)	288 (86.5)	0.36
History of CVD (%)	190 (15.5)	137 (15.3)	53 (15.9)	0.86
Intensive Lifestyle Intervention (%)	618 (50.3)	386 (43.1)	232 (69.7)	<0.001

Data are n (%) or mean (SD). PA: physical activity; DM, diabetes mellitus; BMI, body mass index; SBP, systolic blood pressure; DBP, diastolic blood pressure; FPG, fasting plasma glucose; HbA1c, glycosylated haemoglobin; LDL-c, low-density lipoprotein cholesterol; HDL-c, high-density lipoprotein cholesterol; VLDL-c, very-low-density lipoprotein cholesterol; CVD, cardiovascular disease. Other ethnicities include American Indians and Asian Americans.

© 2024 Huang Z et al. *JAMA Network Open.*

eTable 6. Risk of Primary and Secondary Outcomes Stratified by Only Weight Loss Categories or PA Trajectory

Group	N (%)	Model 1	*P*	Model 2	*P*
**Primary outcome:** CV death, nonfatal MI or stroke, or admission to hospitalization for angina.					
Weight Loss < 7%	144 (16.1)	Reference	-	Reference	-
Weight Loss ≥ 7%	54 (16.2)	0.97 (0.71-1.32)	0.827	0.87 (0.63-1.21)	0.412
Low PA volume	151 (16.9)	Reference	-	Reference	-
High PA volume	47 (13.9)	0.65 (0.47-0.92)	0.014	0.78 (0.55-1.12)	0.177
**Secondary outcome 1:** CV death, non-fatal MI or stroke.					
Weight Loss < 7%	90 (10.0)	Reference	-	Reference	-
Weight Loss ≥ 7%	40 (12.0)	1.16 (0.80-1.69)	0.426	1.16 (0.78-1.73)	0.450
Low PA volume	102 (11.4)	Reference	-	Reference	-
High PA volume	28 (8.3)	0.60 (0.39-0.92)	0.02	0.76 (0.48-1.19)	0.231
**Secondary outcome 2:** all-cause death, non-fatal MI or stroke, or admission to hospitalization for angina.					
Weight Loss < 7%	164 (18.3)	Reference	-	Reference	-
Weight Loss ≥ 7%	70 (21.0)	1.09 (0.82-1.44)	0.547	1.01 (0.75-1.36)	0.946
Low PA volume	182 (20.4)	Reference	-	Reference	-
High PA volume	52 (15.4)	0.60 (0.44-0.83)	0.002	0.71 (0.51-0.99)	0.042
**Secondary outcome 3:** Secondary outcome 2 or CABG, PCI, hospitalization for heart failure, carotid endarterectomy, or peripheral vascular disease.					
Weight Loss < 7%	206 (23.0)	Reference	-	Reference	-
Weight Loss ≥ 7%	78 (23.4)	0.95 (0.73-1.23)	0.677	0.88 (0.67-1.16)	0.364
Low PA volume	222 (24.9)	Reference	-	Reference	-
High PA volume	62 (18.3)	0.59 (0.44-0.78)	<0.001	0.65 (0.48-0.89)	0.006

Model 1 was adjusted for age, race, sex. Model 2 was adjusted for age, race, sex, history of cardiovascular disease, fasting plasma glucose, history of hypertension, insulin use, aspirin use, sedentary time, drinking status, smoking status, triglyceride and treatment arm. CV, cardiovascular; MI, myocardial infarction; CABG, coronary artery bypass grafting; PCI, percutaneous coronary intervention.

© 2024 Huang Z et al. *JAMA Network Open.*

eTable 7. Risk of Primary and Secondary Outcomes Stratified by Weight Loss and MVPA Trajectories Using the Criteria of the American Diabetes Association

Group	N (%)	Model 1	*P*	Model 2	*P*
**Primary outcome:** CV death, nonfatal MI or stroke, or admission to hospitalization for angina.					
Low PA & No Weight Loss	93 (15.8)	Reference	-	Reference	-
Only High PA	34 (17.3)	0.91 (0.61-1.36)	0.64	1.13 (0.75-1.72)	0.56
Only Weight Loss	58 (19.3)	1.23 (0.89-1.72)	0.21	1.12 (0.79-1.58)	0.54
High PA & Weight Loss	13 (9.2)	0.44 (0.24-0.79)	0.006	0.47 (0.26-0.86)	0.01
**Secondary outcome 1:** CV death, non-fatal MI or stroke.					
Low PA & No Weight Loss	59 (10.0)	Reference	-	Reference	-
Only High PA	20 (10.2)	0.86 (0.51-1.44)	0.56	1.11 (0.65-1.91)	0.70
Only Weight Loss	43 (14.3)	1.43 (0.96-2.13)	0.08	1.43 (0.94-2.17)	0.09
High PA & Weight Loss	8 (5.6)	0.45 (0.21-0.95)	0.04	0.56 (0.26-1.21)	0.14
**Secondary outcome 2:** all-cause death, non-fatal MI or stroke, or admission to hospitalization for angina.					
Low PA & No Weight Loss	106 (18.0)	Reference	-	Reference	-
Only High PA	35 (17.9)	0.83 (0.56-1.22)	0.34	1.01 (0.67-1.51)	0.96
Only Weight Loss	76 (25.2)	1.42 (1.05-1.91)	0.02	1.34 (0.98-1.83)	0.07
High PA & Weight Loss	17 (12.0)	0.50 (0.30-0.84)	0.009	0.54 (0.32-0.93)	0.03
**Secondary outcome 3:** Secondary outcome 2 or CABG, PCI, hospitalization for heart failure, carotid endarterectomy, or peripheral vascular disease.					
Low PA & No Weight Loss	135 (22.9)	Reference	-	Reference	-
Only High PA	42 (21.4)	0.77 (0.54-1.10)	0.15	0.88 (0.61-1.27)	0.49
Only Weight Loss	87 (28.9)	1.24 (0.94-1.62)	0.13	1.15 (0.86-1.53)	0.35
High PA & Weight Loss	20 (14.1)	0.45 (0.28-0.73)	0.001	0.47 (0.29-0.77)	0.003

Model 1 was adjusted for age, race, sex. Model 2 was adjusted for age, race, sex, history of cardiovascular disease, fasting plasma glucose, history of hypertension, insulin use, aspirin use, sedentary time, drinking status, smoking status, triglyceride and treatment arm. CV, cardiovascular; MI, myocardial infarction; CABG, coronary artery bypass grafting; PCI, percutaneous coronary intervention; PA, physical activity.

© 2024 Huang Z et al. *JAMA Network Open.*

eTable 8. Risk of Primary and Secondary Outcomes Stratified by Weight Loss and MVPA Trajectories Using MVPA Accumulated in 1-Minute Bouts

Group	N (%)	Model 1	*P*	Model 2	*P*
**Primary outcome:** CV death, nonfatal MI or stroke, or admission to hospitalization for angina.					
Low PA & No Weight Loss	128 (16.1)	Reference	-	Reference	-
Only High PA	16 (16.0)	0.75 (0.44-1.26)	0.28	0.85 (0.50-1.47)	0.57
Only Weight Loss	52 (18.2)	1.11 (0.81-1.54)	0.51	0.99 (0.71-1.39)	0.97
High PA & Weight Loss	2 (4.2)	0.18 (0.04-0.72)	0.02	0.19 (0.05-0.77)	0.02
**Secondary outcome 1:** CV death, non-fatal MI or stroke.					
Low PA & No Weight Loss	80 (10.1)	Reference	-	Reference	-
Only High PA	10 (10.0)	0.80 (0.41-1.55)	0.51	0.98 (0.49-1.95)	0.94
Only Weight Loss	39 (13.7)	1.37 (0.93-2.01)	0.11	1.33 (0.89-2.00)	0.16
High PA & Weight Loss	1 (2.1)	0.15 (0.02-1.09)	0.06	0.19 (0.03-1.40)	0.10
**Secondary outcome 2:** all-cause death, non-fatal MI or stroke, or admission to hospitalization for angina.					
Low PA & No Weight Loss	147 (18.5)	Reference	-	Reference	-
Only High PA	17 (17.0)	0.70 (0.42-1.17)	0.17	0.78 (0.46-1.32)	0.36
Only Weight Loss	68 (23.9)	1.27 (0.95-1.69)	0.11	1.16 (0.85-1.57)	0.35
High PA & Weight Loss	2 (4.2)	0.15 (0.04-0.62)	0.008	0.16 (0.04-0.66)	0.01
**Secondary outcome 3:** Secondary outcome 2 or CABG, PCI, hospitalization for heart failure, carotid endarterectomy, or peripheral vascular disease.					
Low PA & No Weight Loss	184 (23.1)	Reference	-	Reference	-
Only High PA	22 (22.0)	0.74 (0.47-1.17)	0.20	0.79 (0.50-1.26)	0.33
Only Weight Loss	75 (26.3)	1.09 (0.83-1.43)	0.52	1.01 (0.76-1.33)	0.97
High PA & Weight Loss	3 (6.3)	0.18 (0.06-0.57)	0.003	0.18 (0.06-0.58)	0.004

Model 1 was adjusted for age, race, sex. Model 2 was adjusted for age, race, sex, history of cardiovascular disease, fasting plasma glucose, history of hypertension, insulin use, aspirin use, sedentary time, drinking status, smoking status, triglyceride and treatment arm. CV, cardiovascular; MI, myocardial infarction; CABG, coronary artery bypass grafting; PCI, percutaneous coronary intervention; PA, physical activity.

© 2024 Huang Z et al. *JAMA Network Open.*

eTable 9. Risk of Primary and Secondary Outcomes Stratified by Weight Loss and MVPA Volume Trajectories by Model 6

Group	N (%)	Model 1	*P*	Model 2	*P*
**Primary outcome:** CV death, nonfatal MI or stroke, or admission to hospitalization for angina.					
Low PA & No Weight Loss	113 (16.1)	Reference	-	Reference	-
Only High PA	31 (16.1)	0.78 (0.52-1.17)	0.23	0.98 (0.64-1.48)	0.91
Only Weight Loss	47 (19.5)	1.22 (0.87-1.72)	0.26	1.07 (0.75-1.53)	0.71
High PA & Weight Loss	7 (7.6)	0.34 (0.16-0.73)	0.006	0.38 (0.17-0.83)	0.02
**Secondary outcome 1:** CV death, non-fatal MI or stroke.					
Low PA & No Weight Loss	73 (10.4)	Reference	-	Reference	-
Only High PA	17 (8.9)	0.67 (0.39-1.15)	0.15	0.85 (0.49-1.48)	0.58
Only Weight Loss	35 (14.5)	1.40 (0.93-2.10)	0.11	1.35 (0.88-2.07)	0.16
High PA & Weight Loss	5 (5.4)	0.41 (0.16-1.01)	0.05	0.52 (0.20-1.32)	0.17
**Secondary outcome 2:** all-cause death, non-fatal MI or stroke, or admission to hospitalization for angina.					
Low PA & No Weight Loss	130 (18.5)	Reference	-	Reference	-
Only High PA	34 (17.7)	0.75 (0.51-1.10)	0.14	0.91 (0.61-1.35)	0.65
Only Weight Loss	62 (25.7)	1.39 (1.03-1.89)	0.03	1.26 (0.91-1.74)	0.16
High PA & Weight Loss	8 (8.7)	0.34 (0.16-0.69)	0.003	0.37 (0.18-0.77)	0.008
**Secondary outcome 3:** Secondary outcome 2 or CABG, PCI, hospitalization for heart failure, carotid endarterectomy, or peripheral vascular disease.					
Low PA & No Weight Loss	165 (23.4)	Reference	-	Reference	-
Only High PA	41 (21.4)	0.71 (0.50-1.01)	0.06	0.83 (0.58-1.19)	0.30
Only Weight Loss	69 (28.6)	1.20 (0.90-1.59)	0.21	1.08 (0.81-1.46)	0.59
High PA & Weight Loss	9 (9.8)	0.29 (0.15-0.57)	<0.001	0.31 (0.15-0.61)	0.001

Model 1 was adjusted for age, race, sex. Model 2 was adjusted for age, race, sex, history of cardiovascular disease, fasting plasma glucose, history of hypertension, insulin use, aspirin use, sedentary time, drinking status, smoking status, triglyceride and treatment arm. CV, cardiovascular; MI, myocardial infarction; CABG, coronary artery bypass grafting; PCI, percutaneous coronary intervention; PA, physical activity.

© 2024 Huang Z et al. *JAMA Network Open.*

eTable 10. Risk of Primary and Secondary Outcomes Stratified by Weight Loss and MVPA Volume Trajectories Excluding the Participants Experienced Outcomes Within the First 4 Years (N=1151)

Group	N (%)	Model 1	*P*	Model 2	*P*
**Primary outcome:** CV death, nonfatal MI or stroke, or admission to hospitalization for angina.					
Low PA & No Weight Loss	67 (10.7)	Reference	-	Reference	-
Only High PA	23 (10.6)	0.84 (0.51-1.36)	0.47	0.97 (0.59-1.61)	0.91
Only Weight Loss	24 (11.7)	1.09 (0.68-1.75)	0.71	0.99 (0.61-1.60)	0.97
High PA & Weight Loss	6 (5.8)	0.43 (0.18-0.99)	0.05	0.44 (0.18-1.04)	0.06
**Secondary outcome 1:** CV death, non-fatal MI or stroke.					
Low PA & No Weight Loss	48 (7.7)	Reference	-	Reference	-
Only High PA	13 (6.0)	0.66 (0.35-1.24)	0.20	0.82 (0.43-1.58)	0.56
Only Weight Loss	20 (9.7)	1.28 (0.76-2.17)	0.36	1.27 (0.74-2.19)	0.39
High PA & Weight Loss	5 (4.9)	0.52 (0.21-1.33)	0.17	0.64 (0.24-1.67)	0.36
**Secondary outcome 2:** all-cause death, non-fatal MI or stroke, or admission to hospitalization for angina.					
Low PA & No Weight Loss	83 (13.3)	Reference	-	Reference	-
Only High PA	27 (12.4)	0.79 (0.51-1.24)	0.31	0.89 (0.56-1.41)	0.63
Only Weight Loss	39 (18.9)	1.42 (0.97-2.09)	0.08	1.33 (0.89-1.98)	0.16
High PA & Weight Loss	7 (6.8)	0.40 (0.18-0.86)	0.02	0.41 (0.18-0.91)	0.03
**Secondary outcome 3:** Secondary outcome 2 or CABG, PCI, hospitalization for heart failure, carotid endarterectomy, or peripheral vascular disease.					
Low PA & No Weight Loss	116 (18.6)	Reference	-	Reference	-
Only High PA	36 (16.6)	0.75 (0.51-1.09)	0.13	0.79 (0.53-1.18)	0.25
Only Weight Loss	46 (22.3)	1.16 (0.82-1.64)	0.39	1.08 (0.76-1.54)	0.68
High PA & Weight Loss	8 (7.8)	0.31 (0.15-0.64)	0.002	0.31 (0.15-0.65)	0.002

Model 1 was adjusted for age, race, sex. Model 2 was adjusted for age, race, sex, history of cardiovascular disease, fasting plasma glucose, history of hypertension, insulin use, aspirin use, sedentary time, drinking status, smoking status, triglyceride and treatment arm. CV, cardiovascular; MI, myocardial infarction; CABG, coronary artery bypass grafting; PCI, percutaneous coronary intervention; PA, physical activity.

© 2024 Huang Z et al. *JAMA Network Open.*

eTable 11. Risk of Primary and Secondary Outcomes Stratified by MVPA Volume Trajectories and Treated Weight Loss as a Continuous Variable

Group	N (%)	Model 1	*P*	Model 2	*P*
**Primary outcome:** CV death, nonfatal MI or stroke, or admission to hospitalization for angina.					
Weight Loss (Per % change)	-	0.99 (0.97-1.01)	0.35	0.98 (0.96-1.00)	0.12
Low PA volume	151 (16.9)	Reference	-	Reference	-
High PA volume	47 (13.9)	0.66 (0.47-0.93)	0.02	0.80 (0.56-1.14)	0.22
P for interaction	-	-	0.01	-	0.02
**Secondary outcome 1:** CV death, non-fatal MI or stroke.					
Weight Loss (Per % change)	-	1.00 (0.98-1.03)	0.81	1.00 (0.98-1.03)	0.80
Low PA volume	102 (11.4)	Reference	-	Reference	-
High PA volume	28 (8.3)	0.60 (0.39-0.92)	0.02	0.76 (0.48-1.19)	0.23
P for interaction	-	-	0.04	-	0.07
**Secondary outcome 2:** all-cause death, non-fatal MI or stroke, or admission to hospitalization for angina.					
Weight Loss (Per % change)	-	1.01 (0.99-1.03)	0.36	1.00 (0.99-1.02)	0.63
Low PA volume	182 (20.4)	Reference	-	Reference	-
High PA volume	52 (15.4)	0.59 (0.43-0.82)	0.001	0.70 (0.50-0.98)	0.04
P for interaction	-	-	0.003	-	0.003
**Secondary outcome 3:** Secondary outcome 2 or CABG, PCI, hospitalization for heart failure, carotid endarterectomy, or peripheral vascular disease.					
Weight Loss (Per % change)	-	1.00 (0.98-1.02)	0.88	1.00 (0.98-1.01)	0.61
Low PA volume	222 (24.9)	Reference	-	Reference	-
High PA volume	62 (18.3)	0.59 (0.44-0.79)	<0.001	0.66 (0.49-0.89)	0.007
P for interaction	-	-	0.001	-	0.003

Model 1 was adjusted for age, race, sex. Model 2 was adjusted for age, race, sex, history of cardiovascular disease, fasting plasma glucose, history of hypertension, insulin use, aspirin use, sedentary time, drinking status, smoking status, triglyceride and treatment arm. CV, cardiovascular; MI, myocardial infarction; CABG, coronary artery bypass grafting; PCI, percutaneous coronary intervention; PA, physical activity.

© 2024 Huang Z et al. *JAMA Network Open.*

eTable 12. Multivariable Joint Models for Longitudinal Evaluation of Logarithmic Transformation of (PA + 1) and Primary Outcome Including Participants With Baseline and at Least 1 Follow-Up PA Measures

Model	Variables	HR (95%CI)	*P*	*P* for interaction	R hat
JM (basic)	Weight loss	0.91 (0.65-1.29)	0.62	-	1.01
	Log (PA + 1)	0.87 (0.79-0.95)	0.003		1.01
JM (value)*	Weight loss	1.39 (0.72-2.76)	0.32	0.13	1.03
	Log (PA + 1)	0.91 (0.82-1.00)	0.05		1.01
JM (slope)*	Weight loss	0.68 (0.18-2.73)	0.54	0.13	1.22
	Log (PA + 1)	0.03 (0-1.57)	0.09		2.40
JM (time, linear)†	Weight loss	0.93 (0.66-1.31)	0.70		1.00
	Log (PA + 1)	1.02 (0.84-1.24)	0.90	0.06	1.03
JM (time, ns)†	Weight loss	0.94 (0.66-1.33)	0.73		1.00
	Log (PA + 1)	1.05 (0.79-1.48)	0.78		1.23
	Log (PA + 1), 0	0.96 (0.68-1.31)	-	0.84	1.08
	Log (PA + 1), 1	0.67 (0.33-1.14)	-	0.15	1.29
	Log (PA + 1), 4	0.76 (0.56-0.99)	-	0.04	1.02

Participants that completed baseline PA with any or both PA measurements at Year 1 and 4 were included (n=1834), and 290 (15.8%) events were observed. Time-varying effects were conducted using the linear mixed-effects and natural cubic spline models. * P for interaction was calculated by adding multiplicative interaction terms of weight loss and Log (PA + 1). †P for interaction was calculated by adding multiplicative interaction terms of time and Log (PA + 1). PA, physical activity; HR, hazard ratio; JM, joint model; ns, natural cubic splines.

eFigure 1. Flowchart for Participant Selection

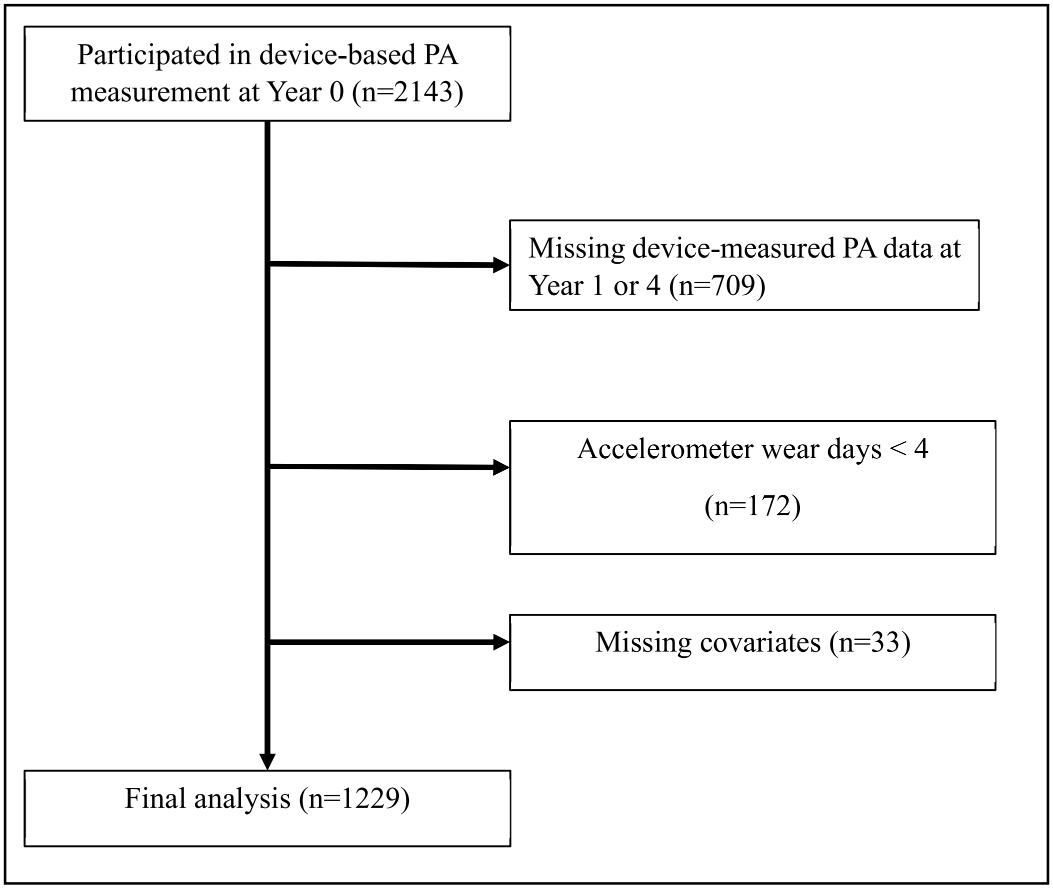

© 2024 Huang Z et al. *JAMA Network Open.*

eFigure 2. Moderate-to-Vigorous Physical Activity Volume Trajectory Model Establishment

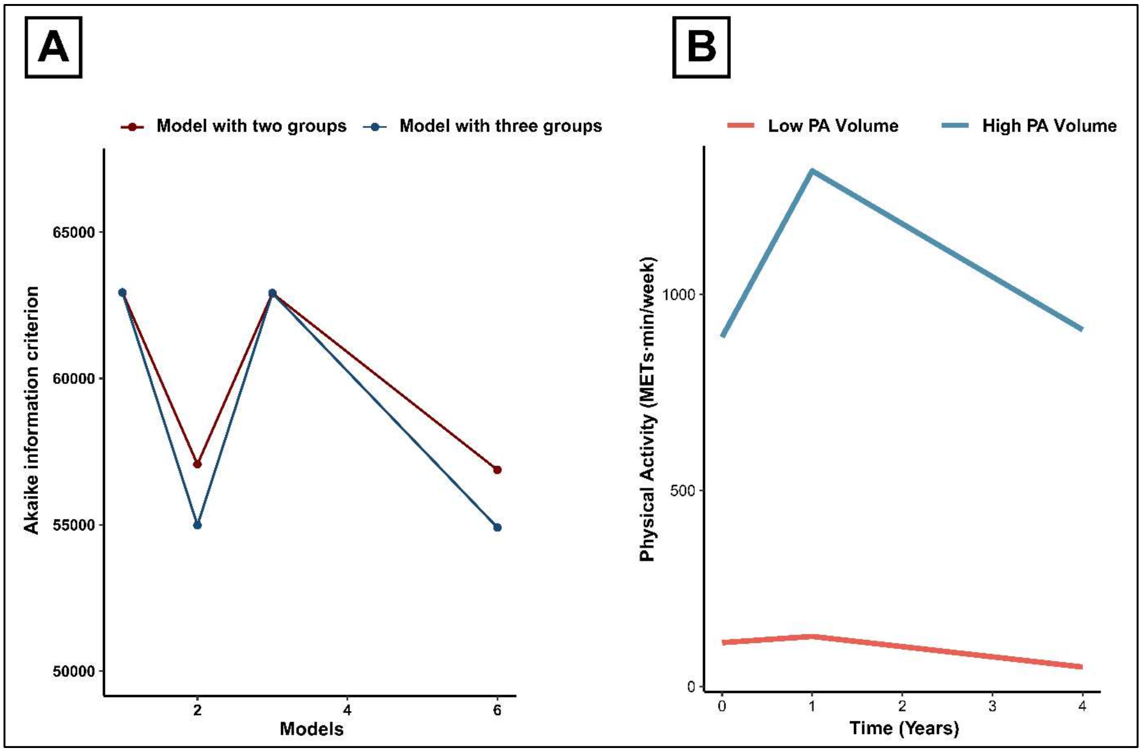

Figure 2A. Comparison of the Akaike information criterion (AIC) of various models. Model with two or three groups in model 2 and 6 had lower AIC values and were hence selected as the potential model of choices. Figure 2B. Moderate-to-vigorous physical activity volume trajectory in the present study. PA, physical activity.

© 2024 Huang Z et al. *JAMA Network Open.*

eFigure 3. Sankey Diagram of 4-Year Body Weight Stratified by Treatment Groups

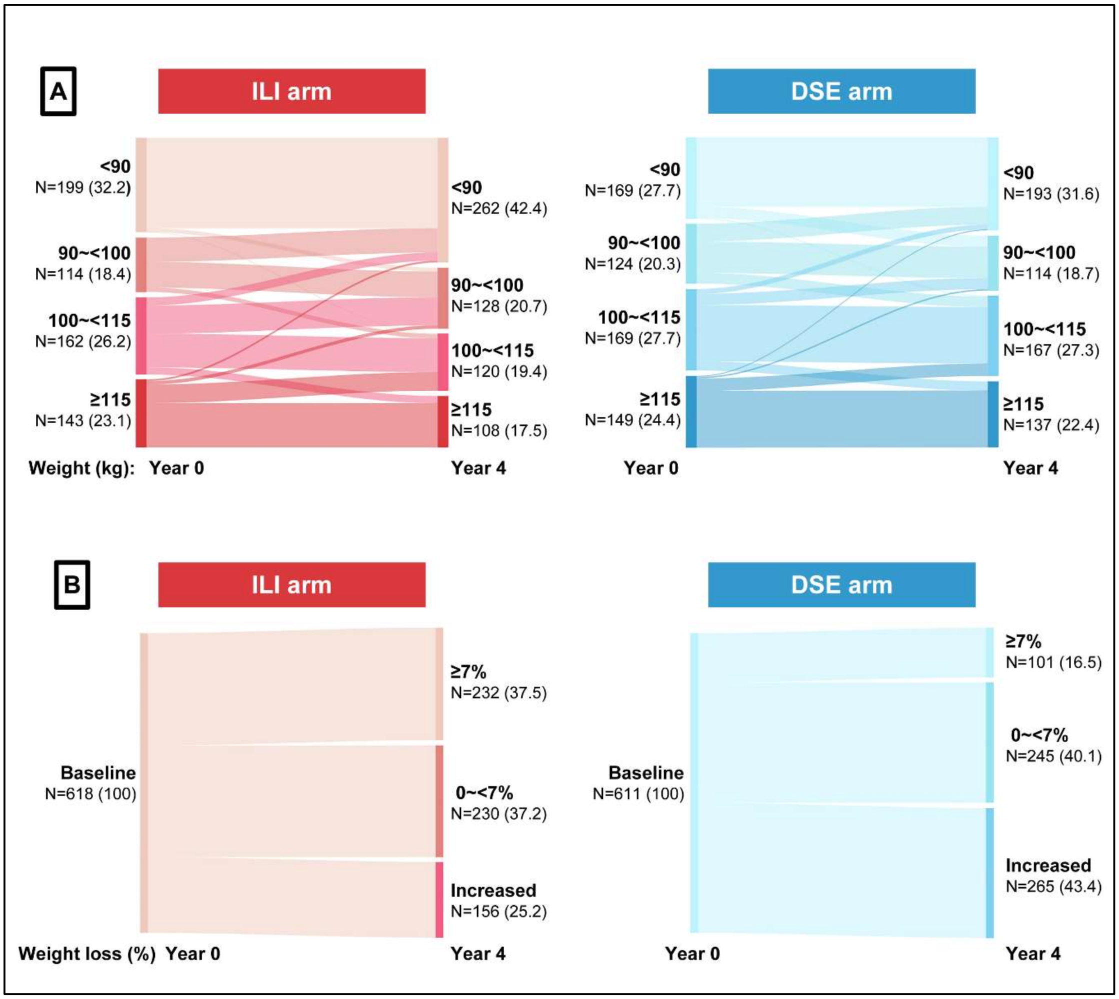

A. Sankey diagrams depicting the change in body weight in ILI and DSE arm. B. Sankey diagrams depicting the 4-year weight loss of baseline body weight in ILI and DSE arm. Figure 1A & B weight loss were attained by participants in both arms. DSE, diabetes support and education; ILI, intensive lifestyle intervention.

© 2024 Huang Z et al. *JAMA Network Open.*

eFigure 4. Cumulative Incidence Estimates for the Primary Outcome and 3 Secondary Outcomes Among Participants Stratified by Weight Loss Categories

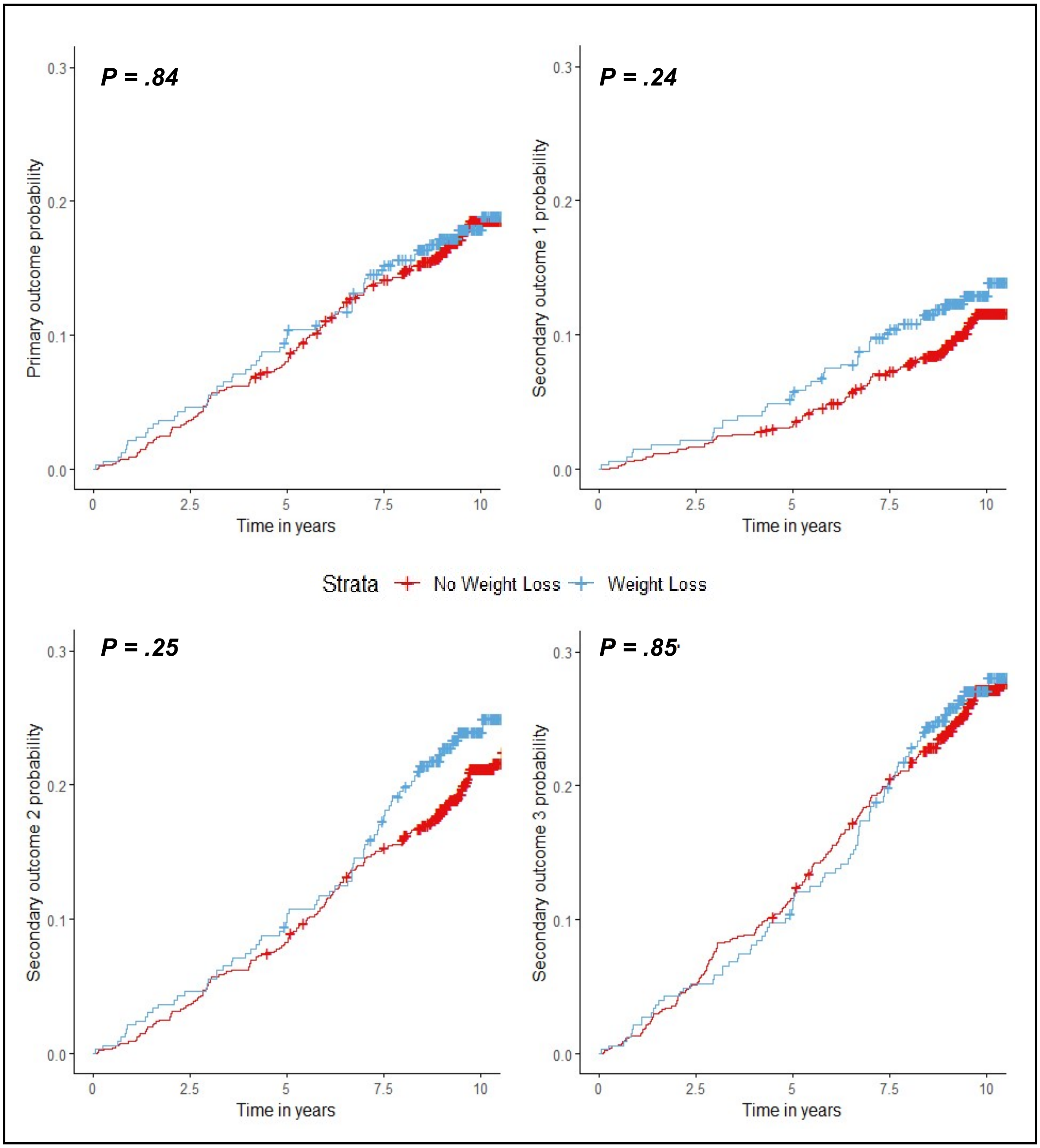

© 2024 Huang Z et al. *JAMA Network Open.*

eFigure 5. Cumulative Incidence Estimates for the Primary and Secondary Outcomes Stratified by Weight Loss Categories and PA Trajectories

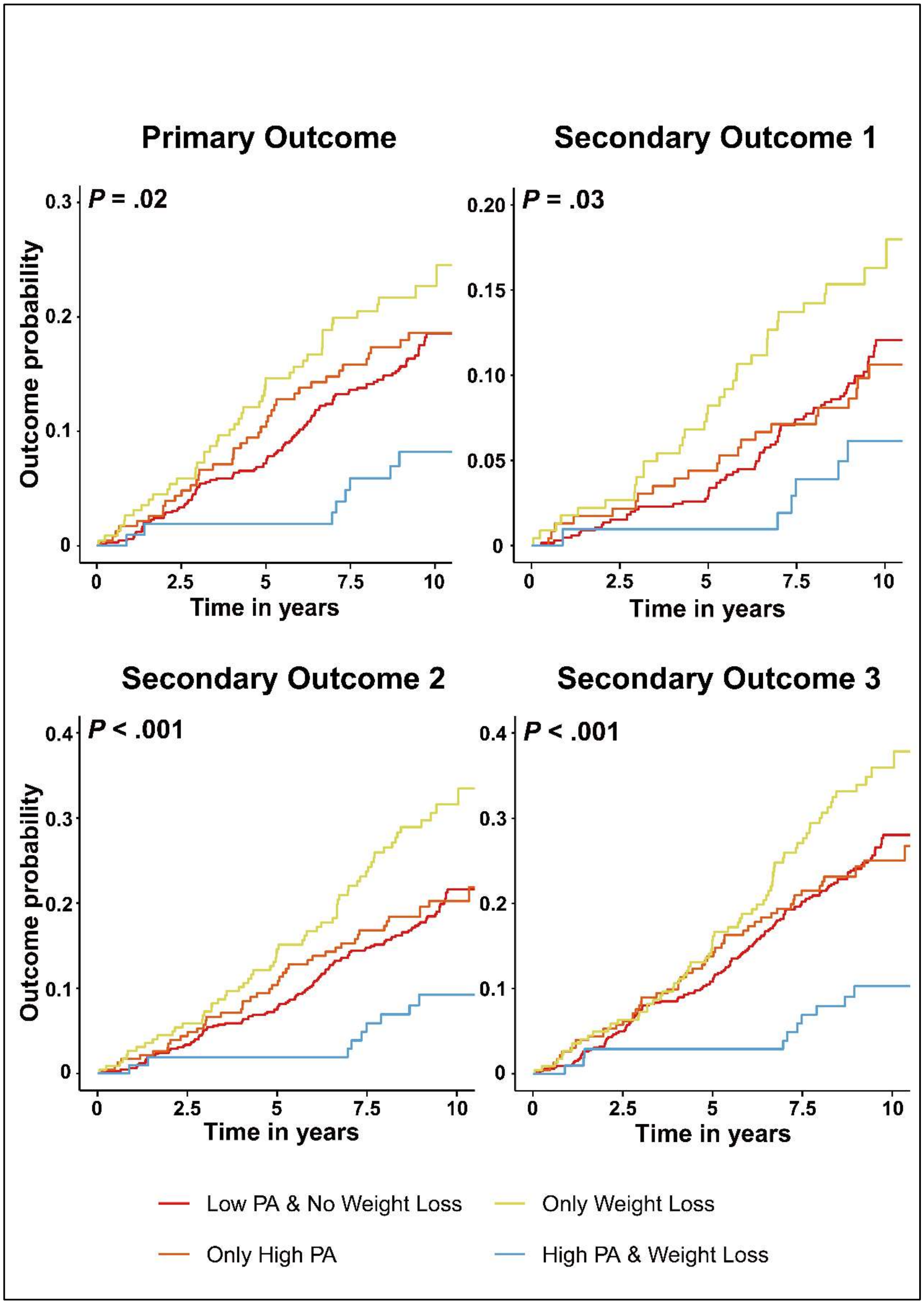

PA, physical activity.

© 2024 Huang Z et al. *JAMA Network Open.*

eFigure 6. Associations Between 4-Year Accumulative Mean PA Volume With Secondary Outcomes Stratified by Weight Loss Categories

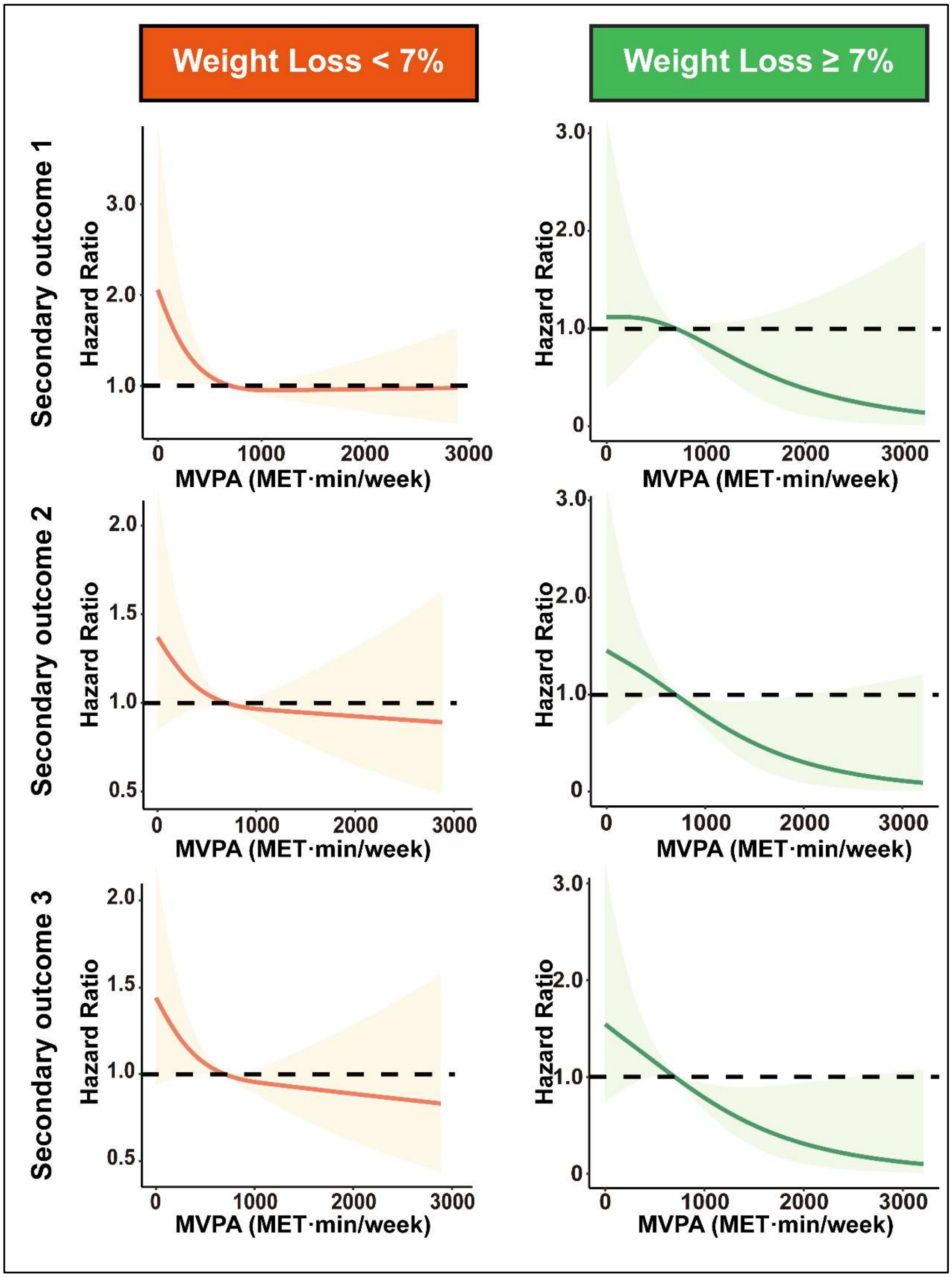

Association was estimated by the restricted cubic splines models. Y-axis indicates the hazard ratios of secondary outcomes. The orange and green ribbons indicate the 95% confident interval. Reference was set at 700 MET·min/week. MVPA, moderate-to-vigorous physical activity.

© 2024 Huang Z et al. *JAMA Network Open.*

eFigure 7. Time-Varying Associations Between PA Volume and Primary Outcomes Stratified by Weight Loss Including Participants With at Least 1 Follow-Up PA Data

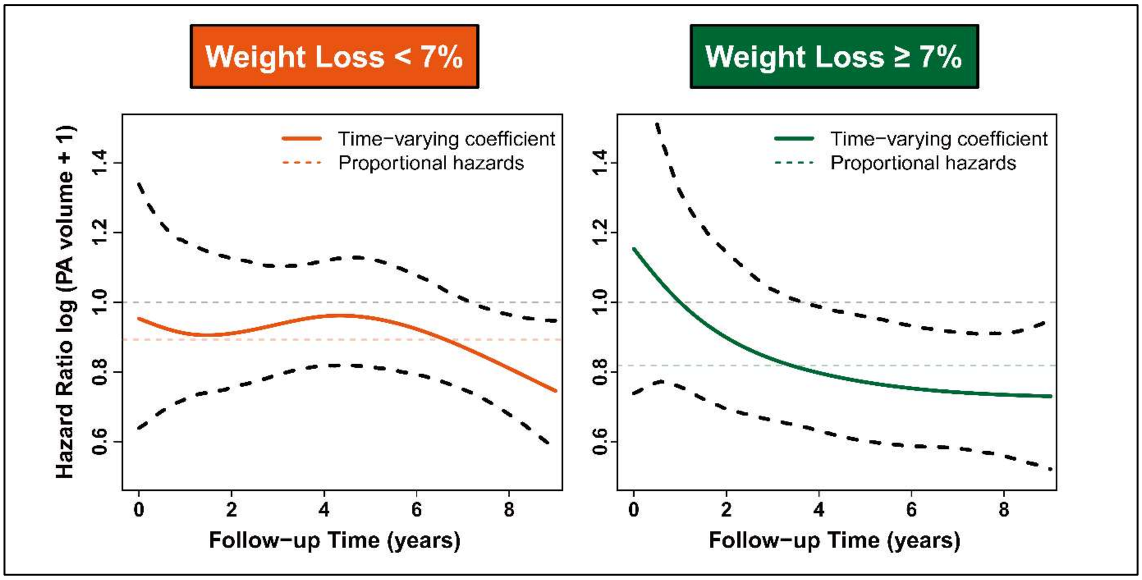

Participants that completed baseline PA and any or both PA measurements at Year 1 and 4 were included (n=1834), and 290 (15.8%) events were observed. Association was estimated by the natural spline models. Y-axis indicates the hazard ratios of Log (PA volume + 1) and primary outcomes over time. The orange and green dash lines indicated the proportional hazard ratio of Log (PA volume + 1). The black dash lines indicated the 95% confidential intervals. The grey dash lines indicated the reference level (hazard ratio = 1.0). PA, physical activity.

© 2024 Huang Z et al. *JAMA Network Open.*

eFigure 8. Subgroup Analysis of the Risk of Primary Outcome

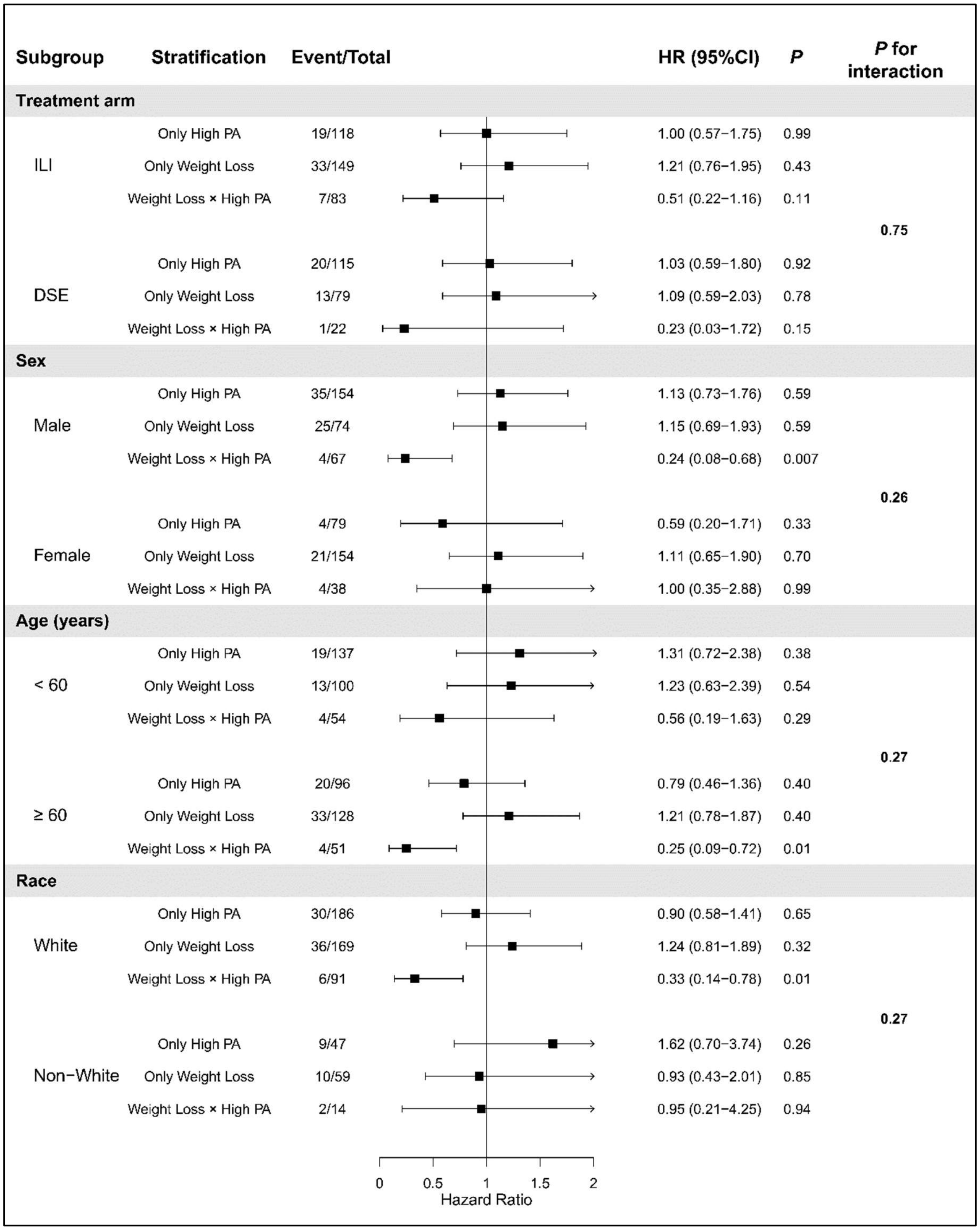

Non-white included the non-Hispanic, Black Hispanic and Other/Mixed. DSE, diabetes support & education; ILI, intensive lifestyle intervention; PA, physical activity.

© 2024 Huang Z et al. *JAMA Network Open.*

eReferences.

1. Ryan DH, Espeland MA, Foster GD, et al. Look AHEAD (Action for Health in Diabetes): design and methods for a clinical trial of weight loss for the prevention of cardiovascular disease in type 2 diabetes. *Controlled clinical trials.* Oct 2003;24(5):610-628. doi: 10.1016/s0197-2456(03)00064-3.
2. Look ARG, Wing RR, Bolin P, et al. Cardiovascular effects of intensive lifestyle intervention in type 2 diabetes. *The New England journal of medicine.* Jul 11 2013;369(2):145-154. doi: 10.1056/NEJMoa1212914.
3. Sampasa-Kanyinga H, Colman I, Goldfield GS, et al. Combinations of physical activity, sedentary time, and sleep duration and their associations with depressive symptoms and other mental health problems in children and adolescents: a systematic review. *The international journal of behavioral nutrition and physical activity.* Jun 5 2020;17(1):72. doi: 10.1186/s12966-020-00976-x.
4. Quan M, Xun P, Wu H, et al. Effects of interrupting prolonged sitting on postprandial glycemia and insulin responses: A network meta-analysis. *J Sport Health Sci.* Jul 2021;10(4):419-429. doi: 10.1016/j.jshs.2020.12.006.
5. Nagin DS, Odgers CL. Group-based trajectory modeling in clinical research. *Annual review of clinical psychology.* 2010;6:109-138. doi: 10.1146/annurev.clinpsy.121208.131413.
6. Desjardins C, Bulut O. profileR: An R package for profile analysis. *Journal of Open Source Software.* 2020;5(47). doi: 10.21105/joss.01941.
7. ElSayed NA, Aleppo G, Aroda VR, et al. 5. Facilitating Positive Health Behaviors and Well-being to Improve Health Outcomes: Standards of Care in Diabetes-2023. *Diabetes Care.* Jan 1 2023;46(Supple 1):S68-S96. doi: 10.2337/dc23-S005.
8. ElSayed NA, Aleppo G, Aroda VR, et al. 8. Obesity and Weight Management for the

© 2024 Huang Z et al. *JAMA Network Open.*

Prevention and Treatment of Type 2 Diabetes: Standards of Care in Diabetes-2023. *Diabetes Care.* Jan 1 2023;46(Suppl 1):S128-S139. doi: 10.2337/dc23-S008.

9. Baart SJ, van der Palen RLF, Putter H, et al. Joint Modeling of Longitudinal Markers and Time-to-Event Outcomes: An Application and Tutorial in Patients After Surgical Repair of Transposition of the Great Arteries. *Circ Cardiovasc Qual Outcomes.* Nov 2021;14(11):e007593. doi: 10.1161/CIRCOUTCOMES.120.007593.

© 2024 Huang Z et al. *JAMA Network Open.*
