# Supplementary material for: Physical Activity and Weight Loss Among Adults With Type 2 Diabetes and Overweight or Obesity: A Post Hoc Analysis of the Look AHEAD Trial
Source: JAMA Netw Open. 2024 Feb 22;7(2):e240219. doi: 10.1001/jamanetworkopen.2024.0219 (PMC10884882; doi:10.1001/jamanetworkopen.2024.0219)
Supplement: Supplement 2. — Data Sharing Statement [file jamanetwopen-e240219-s002.pdf]

Data Sharing Statement

Huang. Physical Activity and Weight Loss Among Adults With Type 2 Diabetes and Overweight or Obesity. *JAMA Netw Open*. Published February 22, 2024.
doi:10.1001/jamanetworkopen.2024.0219

Data
Data available: No
